# Tversky and Kahneman’s Cognitive Illusions: Who Can Solve Them, and Why?

**DOI:** 10.3389/fpsyg.2021.584689

**Published:** 2021-04-12

**Authors:** Georg Bruckmaier, Stefan Krauss, Karin Binder, Sven Hilbert, Martin Brunner

**Affiliations:** ^1^School of Education, Institute of Secondary Education, University of Applied Sciences and Arts Northwestern Switzerland, Windisch, Switzerland; ^2^Mathematics Education, Faculty of Mathematics, University of Regensburg, Regensburg, Germany; ^3^Institute for Learning and Teaching Research, Faculty of Psychology, Education and Sports Science, University of Regensburg, Regensburg, Germany; ^4^Department of Educational Sciences, Faculty of Human Sciences, University of Potsdam, Potsdam, Germany

**Keywords:** statistical reasoning, logical thinking, cognitive illusion, Monty Hall problem, Wason task, Linda problem, hospital problem, Bayesian reasoning

## Abstract

In the present paper we empirically investigate the psychometric properties of some of the most famous statistical and logical cognitive illusions from the “heuristics and biases” research program by Daniel Kahneman and Amos Tversky, who nearly 50 years ago introduced fascinating brain teasers such as the famous Linda problem, the Wason card selection task, and so-called Bayesian reasoning problems (e.g., the mammography task). In the meantime, a great number of articles has been published that empirically examine single cognitive illusions, theoretically explaining people’s faulty thinking, or proposing and experimentally implementing measures to foster insight and to make these problems accessible to the human mind. Yet these problems have thus far usually been empirically analyzed on an individual-item level only (e.g., by experimentally comparing participants’ performance on various versions of one of these problems). In this paper, by contrast, we examine these illusions as a group and look at the ability to solve them as a psychological construct. Based on an sample of *N* = 2,643 Luxembourgian school students of age 16–18 we investigate the internal psychometric structure of these illusions (i.e., Are they substantially correlated? Do they form a reflexive or a formative construct?), their connection to related constructs (e.g., Are they distinguishable from intelligence or mathematical competence in a confirmatory factor analysis?), and the question of which of a person’s abilities can predict the correct solution of these brain teasers (by means of a regression analysis).

## Introduction

Daniel Kahneman and Amos Tversky demonstrated with numerous examples of what are known as “cognitive illusions” the psychologically, linguistically, and mathematically possible explanations for human error in statistical and logical judgment (Tversky and Kahneman, 1974; Kahneman et al., 1982). The cognitive illusions that they introduced then delivered empirical evidence that people’s reasoning abilities are deficient with respect to the laws of logic and probability. Empirically examined and at this point well-known brain teasers are, for instance, the *Linda problem*, the *hospital problem*, the *Wason selection task*, or typical *Bayesian Reasoning* problems. Newer cognitive illusions like the *Monty Hall problem* appeared on the stage at a later date, adding further empirical evidence demonstrating people’s faulty reasoning strategies. The heuristics and biases program attracted the attention of many researchers from various disciplines (e.g., psychology, mathematics [education], logic, and philosophy) and also greatly influenced important applied domains such as medicine, jurisprudence, and economics as it became clear that even experts in those fields are capable of such logical and statistical fallacies even in their own domains (e.g., in medicine: Garcia-Retamero and Hoffrage, 2013; Binder et al., 2018; in economy: Kahneman and Tversky, 1979; Thaler, 1994; or in law: Hoffrage et al., 2000; Schneps and Colmez, 2013).

In the 1990s a countermovement to the heuristics and biases program was started, which was mainly initiated by the German psychologist Gerd Gigerenzer. In the framework of his research groups’ “enlightening program,” cognitive tools were developed in order to equip people to understand cognitive illusions and statistical brain teasers. The idea behind this research was not to train people in problem-solving prior to presenting a problem but simply to *change the representation* of the presented information. The most famous example of that is to replace probabilities in Bayesian reasoning problems (e.g., “80%”) by so-called natural frequencies (e.g., “8 out of 10”), which leads to substantially better performance by participants (McDowell and Jacobs, 2017). This countermovement eventually led to the formation of two “camps,” one of them developing and implementing “facilitated versions” of cognitive illusions and arguing for the importance of problem representation (e.g., Hoffrage et al., 2002; Hertwig et al., 2008; McDowell et al., 2018), and the other insisting on people’s general deficiencies regarding statistical and logical reasoning (e.g., Kahneman and Tversky, 1996; Pighin et al., 2016).

Notably, all of the above-mentioned famous cognitive illusions are usually studied experimentally on just an individual-item level by cognitive researchers. This was true in the program of Kahneman and Tversky (e.g., Kahneman et al., 1982), but also holds for nearly all authors addressing these brain teasers ever since. Furthermore, this seems to be true regardless of which of the two camps a scholar belongs to. Interestingly, experimental researchers from both camps have yet to investigate whether these cognitive illusions form a (reflexive or formative) psychometric construct (in the following: *cogIll*) in either structure.

At least from a theoretical point of view, there are already approaches for considering such problems together. For instance, Stanovich and West (2000) developed the framework CART (Comprehensive Assessment of Rational Thinking; e.g., Stanovich, 2016), which describes different types of tasks and aims to comprehensively assess rational thinking as clearly distinct from intelligence or corresponding established constructs. CART includes, for example, items of probabilistic and statistical reasoning, scientific reasoning, and probabilistic numeracy. However, it is still “only” a systematic, theoretically based compilation of (several hundred) items to capture reasoning; comprehensive results based on their joint empirical measurement are not yet published—in Stanovich’s words: “Now, that we have the CART, we could, in theory, begin to assess rationality as systematically as we do IQ.” (Stanovich, 2016, p. 32).

**FIGURE 1:**
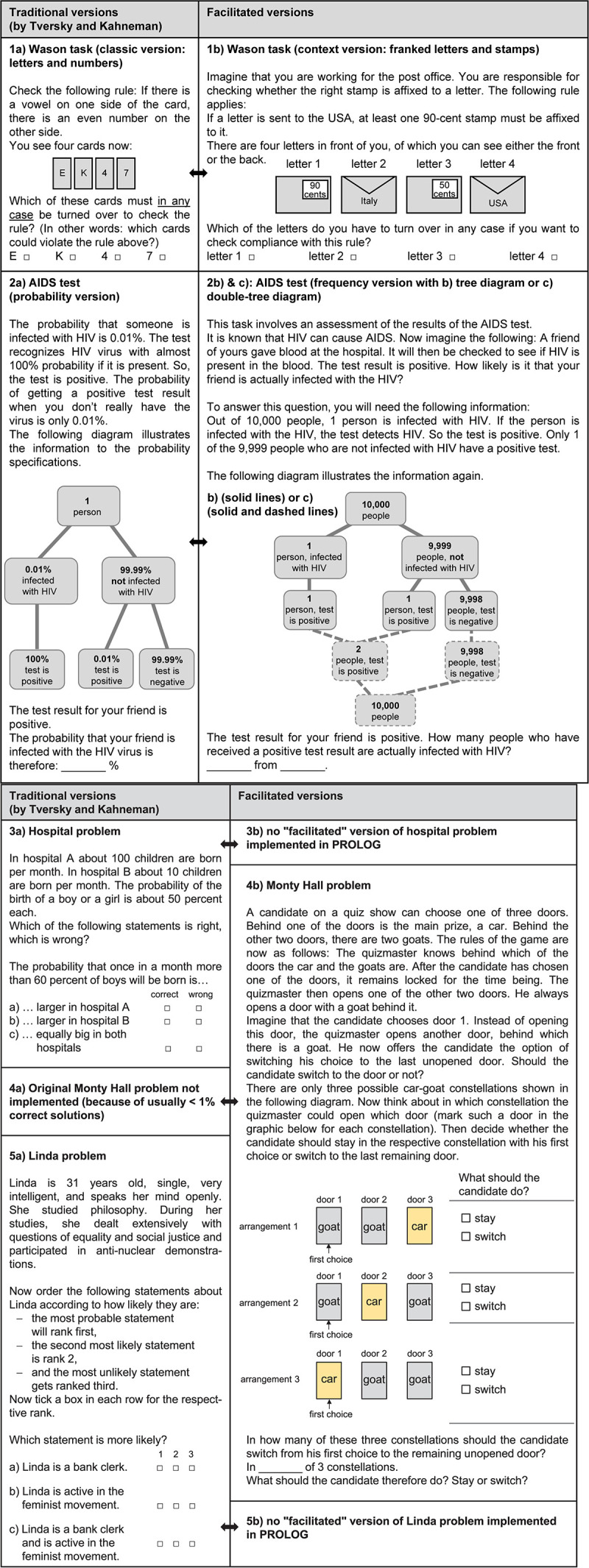
All items of *cogIll*.

In the present study we empirically examine the internal structure of some prominent cognitive illusions (i.e., the most famous ones) when they are considered and implemented simultaneously in one study. The tasks chosen for the present study (see Figure 1) furthermore have the advantage of representing a wide range of problem types and thus entailing a variety of aspects of statistical thinking and logical reasoning.

For example, by means of psychometry we try to answer the question of whether there is a general ability in humans to master such brain teasers or whether the (very few) correct answers given for these problems are rather “random” responses by participants. In addition, we try to explore the relationship of such a supposed ability to seemingly similar competencies like mathematical capacity or general intelligence, and furthermore, whether (and which of) such related capabilities might predict the understanding of statistical and logical brain teasers in regression analyses. By doing so, we will look for possible interactions with respect to the facilitating representations of cognitive illusions mentioned above.

To answer our research questions, we use the data of the large-scale study PISA 2009 in Luxembourg. PISA regularly includes the assessment of mathematics literacy, reading literacy, and intelligence, and in Luxembourg in the year 2009, we were able to supplement tasks in these areas with numerous brain teasers from Tversky and Kahneman’s heuristics and biases program. Thus we not only merge distinguished single cognitive illusions empirically, but also three research traditions theoretically, namely cognitive psychology (here: judgment under uncertainty), teaching of mathematics (here: education of probability theory and statistics), and intelligence research (here: logical and deductive reasoning).

## Theoretical Background

We first unfold the world of Tversky and Kahneman’s heuristics and biases program by presenting examples of concrete illusions (section “Cognitive Illusions From the “Heuristics and Biases” Program (*cogIll*)”), and then theoretically shed light on some established constructs that might come close to *cogIll*, such as mathematical ability or intelligence (section “Person-Related and Task-Related Characteristics Associated With the Ability to Solve Cognitive Illusions”).

### Cognitive Illusions From the “Heuristics and Biases” Program (*cogIll*)

In the following, we present the “traditional” versions of five famous brain teasers that were also addressed in our study (the versions finally implemented in the present study can be found in Figure 1). The names of the problems in the headings will each be followed by the respective logical or statistical concept (in parentheses) that was identified as being difficult to grasp with human intuitive thinking. Regarding each single cognitive illusion, we present and explain the correct solution (including reporting typical solution rates), describe the underlying faulty heuristic that most people follow (according to Kahneman and Tversky), summarize corresponding subsequent research findings, and introduce—if available—didactic tools that can be used to make the original tasks easier to understand.

#### Wason Task (Logical Implication)

The “Wason selection task” is a logical problem containing four cards and one rule. Its traditional version reads as follows (cf. Wason, 1968; for the versions implemented see Figure 1):

You see four cards showing the signs or symbols A, K, 4, and 7 on the front side of the cards. The experimenter claims: “If there is a vowel on one side of the card, then there is an even number on the other side.”

The experimenter then asks: “Which card(s) must be turned over to check whether the rule applies?”

In order to check the rule, the cards showing the A and the 7 (but *not* the 4) have to be turned, since only these cards have the potential to violate the rule (see below). Originally introduced by Wason (1966), his selection task—also called the “Wason card-sorting problem”—has been the subject of dozens of empirical studies since then (e.g., Cosmides, 1989; Evans and Over, 1996; Johnson-Laird, 1999; West et al., 2008; Fiddick and Erlich, 2010; Fitelson and Hawthorne, 2010).

The reason for the enormous interest in this task is that barely 10% of Wason’s participants came up with the right solution to this seemingly simple problem. Of the 128 students to whom Wason first posed this problem, only five gave the correct answer. 46% of the students wanted to turn A and 4, and 33% gave just A as the answer. Indeed, it is usually clear to everyone that the card showing A has to be turned: if there were an odd number on the other side, the rule would be violated. Turning the 4, however, is unnecessary, since even a consonant on the other side would not violate the rule (note that the rule says nothing about the back side of consonants). Yet it is crucial to look at the back side of the card with the 7 because, if there were a vowel on the other side, the rule would be violated, too.

The problem involves reasoning as to how an “if-A-then-B” statement can be falsified (cf. West et al., 2008). Logically, this rule corresponds to the so-called *contraposition law*, meaning that the implications “If A, then B” and “If *not* B, then *no*t A” are equivalent to each other (and thus, only the conditions “A” and “not B” have the capacity to violate the rule). Not only is the correct response to Wason’s selection task usually given by very few participants, but Wason (1968) noticed that when he tried to convince participants of their errors, he encountered unexpected resistance. Interestingly, even when he asked them to turn the card with the 7, and they discovered an A on the other side, they claimed that choosing the 7 was unnecessary.

One cognitive explanation for this error is that most people tend to want to confirm their assumptions with new information rather than try to refute them. Whoever turns card A has the possibility of confirming the rule “if vowel, then even number,” while whoever turns card 7 can at most refute it. There are multiple instances of confirmation biases in the literature, according to which such tendencies are deeply human. Since then, these tendencies have even been proposed to be responsible for belief in pseudosciences and conspiracy theories (cf. Shermer, 2002; Majima, 2015).

The solution rate for the Wason task can be significantly increased, however, by replacing the abstract signs or symbols on the cards with real-world contextualizations, for example by displaying franked letters with different destinations where it is necessary to find out whether a specific franking rule is correctly applied (see Figure 1, right-hand side above). With respect to the contraposition law, it becomes intuitively evident when, for instance, considering the following true, real-world implication: “If I am standing on the Tower Bridge, I must be in London.” The corresponding reverse (and also true) implication is then: “If I am *not* in London, I can*not* be on the Tower Bridge.” Such concrete contextualizations allow even very young students to intuitively grasp the logic behind the rule and to solve analog tasks correctly (e.g., compare the “cheating detection paradigm”; Fiddick et al., 2000; Gummerum and Keller, 2008).

#### Bayesian Reasoning Problems (Inversion of Conditional Probability)

So-called “Bayesian reasoning” problems deal with the inversion of conditional probabilities (well-known examples are, e.g., the cab problem, the AIDS task, or the economics problem). The most famous Bayesian reasoning task is certainly what is known as the “mammography problem” (adapted from Eddy, 1982):

The probability of breast cancer is 1% for a woman of a particular age group who participates in a routine screening. If a woman who participates in a routine screening has breast cancer, the probability is 80% that she will have a positive mammogram. If a woman who participates in a routine screening does not have breast cancer, the probability is 10% that she will have a false-positive mammogram.

What is the probability that a woman of this age group who participates in a routine screening and has a positive mammogram actually has breast cancer?

The correct answer to the question above—about 8%—requires Bayesian reasoning, that is, mathematically inverting the given conditional probabilities in accordance with the formula of Bayes. According to Bayes’ theorem, the resulting *a posteriori* probability p(B| M +) is:

$$p\,\left(B|M+\right)=\frac{p\left(M+|B\right)\cdot p\left(B\right)}{p\left(M+|B\right)\cdot p\left(B\right)+p\left(M+|\neg B\right)\cdot p\left(\neg B\right)}=\frac{80\%\cdot 1\%}{80\%\cdot 1\%+9.6\%\cdot 99\%}\approx 7.8\%$$

The correct result is much lower than most people, even physicians, would expect (Eddy, 1982). The mathematical reason for the counterintuitive low positive predictive value here is the extreme base rate (1%) of the disease that might be neglected by participants (“base-rate neglect”; for alternative explanations see, e.g., Binder et al., 2018; Weber et al., 2018).

Faulty Bayesian reasoning is of high practical relevance. For example, several studies show that even medical doctors (Hoffrage and Gigerenzer, 1998), but patients as well (Garcia-Retamero and Hoffrage, 2013) have difficulties with similar situations. Also, most AIDS counselors, for instance, operate under an illusory belief that positive HIV test results indicate certainty (see Gigerenzer et al., 1998; Ellis and Brase, 2015; Prinz et al., 2015). But in fact, a positive medical test result usually cannot “prove” the presence of a disease. Because sound Bayesian reasoning is not only crucial in the medical domain—inversions of conditional probabilities, for example, are also of relevance in the courts or in the economy—articles on Bayesian reasoning even appear repeatedly in the highly distinguished journals *Science* (Tversky and Kahneman, 1974; Hoffrage et al., 2000; Spiegelhalter et al., 2011; Operskalski and Barbey, 2016) and *Nature* (Goodie and Fantino, 1996).

There are at least two effective strategies that can foster insight into such Bayesian problem situations: (1) translating the statistical information from probabilities (“80%”) into natural frequencies (e.g., “8 out of 10”; Gigerenzer and Hoffrage, 1995; see also Figure 1, right), and/or (2) visualizing the statistical information (for both tools see section “Visualizations”). Meta-analyses confirm the beneficial effect of both measures (McDowell and Jacobs, 2017). A detailed theoretical (psychological and mathematical) discussion on both Bayesian reasoning and natural frequencies can be found in Krauss et al. (2020).

#### Hospital Problem (Empirical Law of Large Numbers)

The so-called “hospital problem” (e.g., Tversky and Kahneman, 1974) is mathematically based on the law of large numbers and reads as follows (cf. Kahneman et al., 1982):

A certain town is served by two hospitals. In the larger hospital about 45 babies are born each day, and in the smaller hospital about 15 babies are born each day. As you know, about 50 percent of all babies are boys. However, the exact percentage varies from day to day. Sometimes it may be higher than 50 percent, sometimes lower.

For a period of 1 year, each hospital recorded the days on which more than 60 percent of the babies born were boys. Which hospital do you think recorded more such days?

The larger hospital

The smaller hospital

About the same (that is, within 5 percent of each other)

Sampling theory entails that the expected number of days on which more than 60 percent of the babies are boys in general is (much) greater in a small hospital than in a large one because a large sample is less likely to stray from 50 percent. More precisely, it follows from the *law of large numbers* that a big sample is more suitable than a small to estimate the parameters of the population (cf. Sedlmeier and Gigerenzer, 1997; West et al., 2008)—although the absolute deviation from the expected value increases the larger the sample is. Interestingly, the mathematician Jacob Bernoulli claimed in 1736 that the law of large numbers is a rule that “even the stupidest man knows by some instinct of nature *per se* and by no previous instruction” (see Gigerenzer et al., 1989, p. 29).

According to Tversky and Kahneman (1974), this fundamental notion of statistics is not a part of people’s repertoire of intuitions. In order to evaluate the probability of obtaining a particular result in a sample drawn from a specified population, people typically rather apply the “representativeness heuristic.” That is, they assess the likelihood of a sample result, for example that the average height in a random sample of ten men will be six feet (183 centimeters), using only the “similarity” of this result to the corresponding parameter (that is, to the average height of, e.g., 180 centimeters in the population of men). Because this similarity does not depend on the size of the sample, people following the representativeness heuristic will ignore sample size. Indeed, when Tversky and Kahneman’s (1974) participants assessed the distributions of average height for samples of various sizes, they produced identical distributions. For example, the probability of obtaining an average height greater than six feet was assigned the same value for samples of 1000, 100, and 10 men. Moreover, their participants failed to appreciate the role of sample size even when it was emphasized in the formulation of the problem.

With respect to the hospital problem, most of Tversky and Kahneman’s participants judged the probability of obtaining more than 60 percent boys to be the same in the small and in the large hospital, presumably because these events are described by the same statistic and are therefore equally representative of the general population (Tversky and Kahneman call it “insensitivity to sample size”). However, surprisingly, the solution rates for the hospital problem have been very different since then. According to Weixler et al. (2019), performances range between 0% (Fischbein and Schnarch, 1997) and 85% (Evans and Dusoir, 1977), the authors attributing the wide range of solution rates to the fact that the tasks used usually varied in one or more features and that the groups of people investigated were different. In disentangling the effects of concrete task and participant characteristics (see below; e.g., grades: Roth et al., 2015; gender: Watson, 2000; see also section “Person-Related and Task-Related Characteristics Associated With the Ability to Solve Cognitive Illusions”), Weixler et al. (2019) found that, for example, problem-solving is facilitated in particular when the deviation from the expected relative frequency is maximal (cf. Lem, 2015), the ratio between the large and the small sample is large (cf. Murray et al., 1987), and/or the order of presented options is “first large, then small sample” (for smaller first: Rubel, 2009, in contrast to the order in Kahneman and Tversky’s, 1972; for other contexts: Fischbein and Schnarch, 1997; Watson and Callingham, 2013). These differences in performance eventually led to contradictory explanations and interpretations of people’s reasoning (in this regard, e.g., Lem et al., 2011).

#### Linda Task (Conjunction Rule for Multiplying Probabilities)

The so-called “Linda task” is based on the conjunction rule for probabilities (cf. Tversky and Kahneman, 1983; Fiedler, 1988; Hertwig and Gigerenzer, 1999):

Linda is 31 years old, single, outspoken, and very bright. She majored in philosophy. As a student, she was deeply concerned with issues of discrimination and social justice, and also participated in anti-nuclear demonstrations.

Which statement is more probable?

(a) Linda is a bank teller (B).
(b) Linda is a bank teller (B) and is active in the feminist movement (F).

The probability of the simultaneous occurrence of two events—for example, p(B∩F)—can be mathematically obtained by multiplying the two involved single probabilities, that is, p(B) ⋅ p(F), or—in the case of the stochastical dependency of B and F—p(B) ⋅ p(F|B). However, the product of two numbers between 0 and 1 always becomes smaller than each of both factors, which is why (a) is the correct option. The description of Linda turns out to be irrelevant here, since it is always more unlikely that two events will happen simultaneously than that only one of both constituents will (thus the content of the events is irrelevant here, too). All that counts are the terms “probability” and “and,” which the conjunction rule interprets, respectively, as mathematical probability and the logical operator “and” (Hertwig, 1995; Gigerenzer and Regier, 1996; Hertwig et al., 2008).

Yet Tversky and Kahneman (1983) found that about 80–90% of participants judged the second option (B∩F) to be more probable than the first option (B). In terms of the heuristics and biases program, the Linda problem is another instance of the representativeness heuristic, since the second option seems to be more representative of Linda than the first. The so-called “conjunction fallacy” in the form of the Linda task or similar problems has also been examined extensively since then (e.g., Fiedler, 1988; Reeves and Lockhart, 1993; Donovan and Epstein, 1997; Hertwig et al., 2008; Wedell and Moro, 2008; Charness et al., 2010). Hertwig and Chase (1998), for instance, found that the proportion of conjunction fallacies could be substantially reduced (from 78% to 42%) by changing the response format from ranking to concrete probability estimation. Interestingly, although there is no concrete probability given, the Linda problem can also be understood more easily using the natural frequency concept introduced in the context of Bayesian reasoning problems (see above). When participants are simply instructed to imagine 200 women who fit Linda’s description, they realize that there must be more women who are bank tellers than women who are both bank tellers and feminists (for details see, e.g., Fiedler, 1988; Hertwig and Gigerenzer, 1999).

#### Monty Hall Problem (Inversion of Conditional Probabilities; Extended Bayesian Reasoning)

The Monty Hall problem (or “three-door problem” or “goat problem”), which had not yet been formulated at the time of Tversky and Kahneman’s first publications but today is one of the most famous examples of a cognitive illusion, is sometimes even considered the “queen” of statistical brain teasers (e.g., Gilovich et al., 2002; Krauss and Wang, 2003; Risen and Gilovich, 2007; Tubau et al., 2015). The traditional formulation of the Monty Hall problem (in the real TV game show, the host Monty Hall played several variations of this setting; see Friedman, 1998) reads as follows:

Suppose you’re on a game show and you’re given the choice of three doors. Behind one door is a car; behind the others, goats. You pick a door, say Number 1, and the host, who knows what’s behind the doors, opens another door, say, Number 3, which has a goat.

He then says to you, “Do you want to switch to Door Number 2?” Is it to your advantage to switch your choice?

The intended rules and conditions of the problem are (e.g., Krauss and Wang, 2003): After the candidate has chosen a door, this door stays locked for the time being. The game show host, who knows behind which door the car is, then opens one of the two remaining doors, which has a goat behind it. Afterward, he offers the player the option of either sticking with his original choice or changing his decision and switching to the other closed door.

Most people think it does not matter whether the candidate changes to the last remaining door or stays with his/her first choice because s/he still has two equally good alternatives to choose from. However, this reasoning ignores the information provided by the open door. Indeed, the probability of winning the car by sticking with the original choice is only 1/3, while the probability of winning by switching to the last remaining door is 2/3. In fact, the mathematical solution to the Monty Hall problem turns out to be a (very) special case of Bayesian reasoning, since the probability that the car is behind Door 2 can be expressed in terms of Bayes’ rule as follows (assuming that the player first chooses Door 1 and that Monty Hall then opens Door 3, which is the standard version):

$$p\,\left(C_{2}|M_{3}\right)=\frac{p\,\left(M_{3}|C_{2}\right)\cdot p\,\left(C_{2}\right)}{p\,\left(M_{3}|C_{1}\right)\cdot p\,\left(C_{1}\right)+p\,\left(M_{3}|C_{2}\right)\cdot p\,\left(C_{2}\right)+p\,\left(M_{3}|C_{3}\right)\cdot p\,\left(C_{3}\right)}=\frac{1\cdot \frac{1}{3}}{\frac{1}{2}\cdot \frac{1}{3}+1\cdot \frac{1}{3}+0\cdot \frac{1}{3}}=\frac{2}{3}$$

where C*_*i*_* = car is located behind door *i*, *i* = 1, 2, 3, and M_3_ = Monty opens Door 3. Note that the solution of course holds regardless of the door specifications given in the standard version.

As with the illusions (1–4) presented thus far, not only do most people misjudge this assessment, but the wrong intuition—“both remaining alternatives have a 50% chance of winning”—often appears to them to be “obvious” (Paley, 2005), and they even dare to offer a higher wager as a result of that belief (vos Savant, 1997).

Many researchers have explored possible reasons for this cognitive fallacy and proposed didactical strategies that could help people to realize the underlying mathematical structure of this situation. For instance, Krauss and Wang (2003) added a frequency question in order to exploit the natural frequency concept, and subsequently Krauss and Atmaca (2004) made the option of a frequency algorithm even more salient by clearly depicting the three possible car-goat constellations (see Figure 1, right). For a recent review of literature addressing why humans systematically fail to react optimally to the Monty Hall problem, see Saenen et al. (2018).

While problems 2–5 theoretically belong to probability theory, problem 1 (the Wason selection task) belongs to the world of logic (note, however, that logic can be considered a restriction of probability theory to the values 0 and 1). In the next section (“Person-Related and Task-Related Characteristics Associated With the Ability to Solve Cognitive Illusions”), we will take a closer look at both individual and task-related characteristics as possible predictors for solving such cognitive illusions.

### Person-Related and Task-Related Characteristics Associated With the Ability to Solve Cognitive Illusions

When research on cognitive illusions began, their generality and their independence from higher education were both praised (e.g., Slovic et al., 1976; Thaler, 1985). For example, Gould (1992) says, “Tversky and Kahneman argue, correctly, I think, that our minds are not built (for whatever reason) to work by the rules of probability” (Gould, 1992, p. 469). And Piattelli-Palmarini (1991) summarizes, “We are a species that is uniformly probability-blind, from the humble janitor to the Surgeon General […]. We should not wait until Tversky and Kahneman receive a Nobel prize^1^ for economics. Our self-deliberation from cognitive illusions ought to start even sooner.”

Yet since then, these considerations and analyses have become more differentiated, and constructs such as numeracy or intelligence have come to be considered covariates in the framework of cognitive illusions. In the following we will discuss factors that might influence performance on statistical and logical cognitive illusions, first at the individual level of participants (sections “Mathematical Competence” to “Further Individual Prerequisites”) and second at the level of the task (sections “Contextualization” to “Visualizations”).

#### Person-Related Prerequisites

Stanovich (2012), for instance, claims that individual differences have largely been ignored in the rationality debate opened up by the heuristics and biases program (also see Evans et al., 1993; Stanovich and West, 1998, 2008). The following *individual* preconditions have thus far been considered as producing variability in responding to brain teasers.

#### Mathematical Competence

Obviously, it is reasonable to assume that mathematical competence might play an essential role in solving cognitive illusions of this kind. And indeed, the relevance of mathematical skills in solving individual brain teasers has already been documented in several studies. For example, Inglis and Simpson (2004, 2005) administered a version of the Wason selection task to three groups, mathematics undergraduates, mathematics academic staff, and history undergraduates (whom Inglis and Simpson chose to represent the general population), finding that both mathematics staff and students were significantly more likely to make the correct selection (and significantly less likely to make the standard mistake). The authors conclude that there is a significant difference between mathematical and non-mathematical cognition. Regarding tasks about the law of large numbers (cf. the hospital problem), even Kahneman and Frederick (2002, p. 50) state that the “mathematical psychologists who participated in the survey not only should have known better—they did know better.”

Regarding Bayesian reasoning, Hill and Brase (2012) examined whether a basic level of numeracy is needed (the so-called “threshold hypothesis”). Although the highly numerate tend to perform better across formats, results are mixed regarding the interaction of the effect of numeracy and the effect of information format (Chapman and Liu, 2009; Hill and Brase, 2012; Johnson and Tubau, 2013). Moreover, Galesic et al. (2009) found that natural frequencies, for instance, can facilitate performance even for individuals with low numerical ability. Finally, regarding the Monty Hall problem, there is evidence that high numeracy level is helpful for recognizing the correct solution after the problem is simulated many times (Lee and Burns, 2015).

#### Reading Competence

Understanding and solving cognitive illusions could also require a certain degree of reading competence. Especially for text-heavy tasks such as typical Bayesian reasoning problems, reading skills might be essential for correctly interpreting the given information. Also, the understanding of logical operators (such as the correct mathematical meaning of “and” in the Linda task; see, e.g., Hertwig et al., 2008) or statements (such as the “if-then structure” in the Wason task; Liu et al., 1996) requires linguistic skills. At the same time, there have also been numerous empirical findings on the influence of text complexity and the tasks’ exact linguistic formulations on solution rates. For example, it has been shown that the complexity and length of the text (Macchi, 2000) and the use of implicit or explicit questions (Böcherer-Linder et al., 2018) can substantially impact solution rates (see also Gigerenzer and Hoffrage, 1999; Mellers and McGraw, 1999; Girotto and Gonzalez, 2002; Johnson and Tubau, 2013).

Moreover, many studies have of course investigated with school students the role of reading skills on mathematics ability in general, where empirical findings also show that students’ mathematical performance is significantly related to general language competence and text comprehension ability (Duarte et al., 2011; Vukovic and Lesaux, 2013; Prediger et al., 2015; Paetsch et al., 2016; Plath and Leiss, 2018). In particular, reading and understanding the text of the task poses problems for many students and can lead to difficulties and errors in the subsequent mathematical task work (Clarkson, 1991; Mayer and Hegarty, 1996; Wijaya et al., 2014). Aside from the basic requirements of the subject of mathematics (i.e., technical terms and academic language), increased verbal complexity in problem presentation was shown to reduce performance (Johnson and Tubau, 2013), suggesting a role for basic text comprehension abilities in performance on Bayesian reasoning problems as well. In an overview, Schleppegrell (2007) synthesizes research by linguists and mathematics educators to highlight the linguistic challenges of mathematics.

#### General Intelligence (Reasoning)

It is very plausible that correctly solving cognitive illusions may depend on general cognitive skills (i.e., *g*). A number of studies—especially from the research group around Stanovich—have shown that individual differences in *g* have been associated with the ability to find normatively correct solutions across a range of decision-making tasks (e.g., Stanovich and West, 2000; Kokis et al., 2002). Some researchers have argued that this is just further evidence of the consistent positive correlations found across diverse measures of abstract cognitive ability (e.g., Hunt, 2000), whereas other researchers (e.g., Stanovich and West, 1998) have suggested that *g* will play the strongest role in abstract or decontextualized forms of reasoning (cf. Kaufman et al., 2011; see also section “Contextualization”). Regarding cognitive illusions in general, Stanovich (2012) argues that there are few consistent individual differences in intuitive, heuristic reasoning, while explicit, knowledge-based reasoning about such tasks may be connected to both crystallized intelligence (i.e., learned knowledge) and fluid intelligence (which is close to *g*). In sum, Stanovich (2012) claims that one should expect a correlation between intelligence and solving cognitive illusions because mindware gaps most often arise from lack of education or experience.

Also, specifically with respect to Bayesian reasoning, empirical evidence is mixed, especially concerning interactions with information format (for details see section “Natural Frequencies”). Regarding tasks in probability format, Stanovich and West (2000) did not find any systematic correlations with cognitive capacity measures (cf. Barbey and Sloman, 2007). On Bayesian tasks in natural frequency format, a higher proportion of correct responses was observed in experiments that selected participants with a higher level of general intelligence as indexed by the academic selectivity of the university the participant attended (Cosmides and Tooby, 1996; Brase et al., 2006). Along the same lines, Sirota et al. (2014) empirically found that cognitive abilities indeed predicted Bayesian performance, especially in the natural frequency format. However, there is also evidence that with respect to Bayesian reasoning tasks, higher general intelligence is linked to improved performance *across* formats (Sirota and Juanchich, 2011; Lesage et al., 2013; McNair, 2015).

According to Stanovich (2012), fluid intelligence reflects reasoning abilities operating across a variety of domains—in particular novel ones. Since it is measured by tasks of abstract reasoning, fluid intelligence will, of course, in some way be related to rationality (here: mastering cognitive illusions) because it indexes the computational power of the algorithmic mind to sustain decoupling. He also argues that individual differences in fluid intelligence are a key indicator of the variability across individuals in the ability to sustain decoupling operations (Stanovich, 2009, p. 353).

Regarding the Monty Hall problem, De Neys and Verschueren (2006) examined whether the notorious difficulty of this special Bayesian task is associated with limitations in working memory resources (which some researchers again equate with *g*). They found that participants who solved the Monty Hall problem correctly had a significantly higher working memory capacity than those who responded erroneously. In addition, correct responding decreased under the mental load of a second parallel task.

#### Further Individual Prerequisites

Other possible personality traits that might also be considered in this context are, for instance, gender, age, educational background (which for students, e.g., is usually measured by the socioeconomic status, SES), and prior experience. The role of gender in mathematics ability has been discussed for decades. Now there are arguments that similarities between the sexes take precedence over differences (e.g., Hyde, 2014). For instance, a meta-analysis shows a large variability in both the size and the direction of gender effects in mathematics performance (Else-Quest et al., 2010; but see Brunner et al., 2008). Concerning stochastics in particular, Engel and Sedlmeier (2005) found no gender difference. Regarding the hospital problem, however, where only a few studies report data on gender at all (e.g., Rasfeld, 2004; Watson and Callingham, 2013), only Watson (2000) explicitly considered gender effects and found very few differences between females and males (in favor of males). Thus there is still a necessity for investigating possible gender differences regarding stochastic tasks in general or cognitive illusions specifically (Roth et al., 2015).

Empirical studies so far provide mixed findings on whether greater age or prior stochastics education (Reagan, 1989) increases solution rates in statistical reasoning in general (e.g., Batanero et al., 1996; Rasfeld, 2004; Brase, 2014; Siegrist and Keller, 2011). However, it was found that the closer the data presented in the task were to self-reported experiences, the more accurate people’s answers were, indicating that the subjective *a priori* estimate (of the probability of a certain event) developed through lived experience had a substantial impact on the reasoning process (Reani et al., 2019).

#### Task-Related Features

In addition to individual factors, of course, characteristics of the *task* play a role with respect to performance as well. In the following, we will explain in detail some “didactical simplifications” of specific cognitive illusions (already briefly addressed above).

#### Contextualization (Wason Selection Task)

Cosmides and Tooby (1992) showed that a change of the abstract rule (i.e., “*p → q*”) in a problem accommodated in a more natural and familiar context than the mere card-checking setup significantly increases the number of correct answers of participants (cf. Besold, 2013). To date, many different modified versions have been used along with the classical abstract problem formulation (e.g., Gigerenzer and Hug, 1992; also see Figure 1, right), for example:

Imagine you are working for the post office. You are responsible for checking whether the right stamp is stuck on a letter. The following rule applies: If a letter is sent to the United States, at least one 90-cent stamp must be stuck on it. There are four letters in front of you, of which you can see either the front or the back (front of letter with “50 cent” and “90 cent,” back of letter with “Italy” and “United States”).

Which of the letters do you have to turn over if you want to check compliance with this rule?

As Gigerenzer and colleagues were able to demonstrate, the solution rate increased substantially with the use of this representation, even though, from the point of view of logic, the situation was unchanged from the original version (Gigerenzer and Hug, 1992; Fiddick et al., 2000). In similar scenarios, even very young people can understand the logic behind a puzzle based on real contexts in the sense of a “cheating detection paradigm” (e.g., “If Maxi cleans up her room, she is allowed to go to the playground,” cf. Gummerum and Keller, 2008). The same holds true in an analogous way for other cognitive illusions. In this respect, the solution rate for Bayesian reasoning tasks, for example, would be even lower if the context were removed and replaced by abstract letters (instead of concrete events) and mathematical symbols, such as “p(A),” etc.

It should be noted that such contextualization in mathematics education research corresponds to the aspect of *modeling* (i.e., considering problems formulated in a real-world context; e.g., Kaiser and Sriraman, 2006). Within this framework, sometimes even previously purely inner-mathematical, abstract tasks are consciously enriched by being related to a reality that is as close as possible to the student’s everyday life in order to make them more accessible and appealing to students (for an overview, see Niss and Blum, 2020).

#### Natural Frequencies (Bayesian Reasoning Tasks)

In a seminal paper, Gigerenzer and Hoffrage (1995) translated the numbers in the breast-cancer screening problem (see section “Cognitive Illusions From the “Heuristics and Biases” Program (*cogIll*)”) into natural frequencies:

Mammography problem (natural frequency format):

100 out of 10,000 women of a particular age group who participate in a routine screening have breast cancer. 80 out of 100 women who participate in a routine screening and have breast cancer will have a positive mammogram. 950 out of 9,900 women who participate in a routine screening and have no breast cancer will have a false-positive mammogram.

How many of the women who participate in a routine screening and receive positive mammograms have breast cancer?

This mode of representation of the statistical information makes it possible to imagine concrete persons; the nested-set relations become transparent, and thus the solution algorithm becomes simpler. Given the natural frequency version, significantly more people are able to make the correct inference (Gigerenzer and Hoffrage, 1995; Siegrist and Keller, 2011) because only the proportion of women with breast cancer among those who have a positive mammogram (i.e., “80 out of 80 + 950” = “80 out of 1,030” = 7.8%) has to be calculated. A meta-analysis by McDowell and Jacobs (2017) summarized 35 studies that implemented natural frequencies and found an average performance increase in such versions of Bayesian reasoning problems of about 24%, compared to only 4% in probability versions.

The concept of natural frequencies can be extended to diagnostic situations with more than one medical test available (Krauss et al., 1999), but it is also applicable to other statistical problems (regarding the Linda problem, e.g., see Fiedler, 1988). In the context of the Monty Hall problem, for instance, a frequency algorithm can be applied to the three possible car-goat constellations (see Figure 1, right-hand side; Krauss and Wang, 2003).

#### Visualizations

Pagin (2019), for instance, investigated the Linda problem by using a task version in which the situation was presented with a Venn diagram. As a consequence, the rate of the conjunction fallacy in a group of participants was substantially lower.

With respect to the Wason task and the corresponding visualizations (see Figure 1 left or right, respectively), Gummerum and Keller (2008) have also successfully worked with pictures of, for example, the (un)tidy room of their protagonist “Maxi” to offer a visualization of the corresponding context.

There are many types of visualizations that can improve Bayesian reasoning, for example, *2* × *2 tables* (e.g., Steckelberg et al., 2004; Binder et al., 2015), *tree diagrams* (e.g., Sedlmeier and Gigerenzer, 2001; Budgett et al., 2016; Bruckmaier et al., 2019), *double-trees* (Khan et al., 2015; Böcherer-Linder and Eichler, 2019), *icon arrays* (e.g., Zikmund-Fisher et al., 2014; contrary findings by Reani et al., 2018), different kinds of *set diagrams* (e.g., *Euler diagram*, or *Venn diagram*; e.g., Reani et al., 2018), *roulette-wheel diagrams* (e.g., Brase, 2014), *frequency grids* (e.g., Garcia-Retamero et al., 2015), *Eikosograms* (also called *unit squares* or *mosaic plots*; e.g., Böcherer-Linder and Eichler, 2017), and *frequency nets* (Binder et al., 2020); for an overview see, for example, Binder et al. (2015).

Regarding the specific Bayesian situation of the Monty Hall problem, the triggering of a counting algorithm by a frequency question (Krauss and Wang, 2003) can be supported by explicitly depicting the three possible car-goat constellations (Krauss and Atmaca, 2004), and thus combining didactic simplifications (see sections “Natural Frequencies” and “Visualizations”) is possible in this case as well.

## The Current Study and Research Questions

In the present study we initially examine, on the basis of the responses of Luxembourgian school students of age 16–18, whether various cognitive illusions (*cogIll*) from Tversky and Kahneman’s heuristics and biases program form a (reflexive or formative) construct in a psychometric sense (RQ 1a). In addition, by means of confirmatory factor analysis, we investigate how such a supposed competence is related to *mathematical literacy* (*ml*) and *intelligence* (*g*) and whether these three abilities are distinct constructs (RQ 1b). Finally, we explore by means of regression models (including Bayesian models) which student abilities and which task characteristics can predict the mastering of cognitive illusions, both at the construct level and in terms of the singular illusions (besides *ml* and *g*, we here include further possible predictors such as *reading literacy* (*rl*), RQ2). In sum:

Research question 1a (reliability and correlational analysis):

Do the tasks of the heuristics and biases program (*cogIll*) form a reflexive or a formative construct? What intercorrelations do individual tasks have and what causes can be found for differential correlations (e.g., What role do facilitations of cognitive illusions play with respect to their mutual correlations)?

Research question 1b (latent confirmatory factor analysis):

Is *cogIll* unidimensional? What is the relationship (i.e., the latent correlations) between *cogIll*, *ml*, and *g*? Can three correlated yet still distinct constructs be corroborated by means of this method?

Research question 2 (regression analysis):

Which abilities and/or task characteristics can predict *cogIll* (or the individual brain teasers)? In addition to the constructs considered in RQ 1b, we will add further predictors like reading literacy here.

## Method

### Design

PROLOG was a study conducted as an *accompanying study* of the Luxembourgian PISA 2009 study (cf. Organisation for Economic Co-operation and Development [OECD], 2010). The key idea was to add famous brain teasers to the PISA scales in order to analyze probabilistic (“PRO”) and logical (“LOG”) thinking as well as their determinants by using a large and representative sample of school students of age 16 (and older).

Note that due to the size of Luxembourg, PISA is a mandatory complete survey for *all* 15-year-old students in the country. Therefore, all 15-year-old students from grade nine and ten must participate, while their younger or older classmates do not have to (the older students usually have no required activity while PISA is administered). Making use of this special situation in Luxembourg, PROLOG was administered to both ninth- and tenth-graders *above* the age of 15 (*N* = 2,643) while their 15-year-old classmates were working on PISA 2009. Note that in order not to endanger the integrity of the actual PISA 2009 study, we implemented items from the PISA 2000 mathematics and reading test in PROLOG.

### Instruments

In the following we describe the items of all constructs implemented.

#### Cognitive Illusions (*cogIll*)

Figure 1 displays all eight cognitive illusions implemented by PROLOG: (1) two versions of the Wason task: (1a) classic version and (1b) facilitated version; (2) three different versions of a Bayesian reasoning problem, namely the AIDS task: (2a) probability version, (2b) frequency version with tree diagram, and (2c) frequency version with double-tree diagram as delineated in Figure 1); (3) the hospital problem; (4) the Linda problem; and (5) the Monty Hall problem. While both versions of the Wason task were provided to the participants simultaneously (i.e., first traditional and then facilitated), only one of the three versions of the AIDS task was presented to each student. The reason for this was that both versions of the Wason task (see Figure 1 on the left for the original and on the right for the contextualized version) seem distinctly different at first sight, in other words, because of the different context not immediately recognizable as basically identical tasks. For the AIDS task, however, the contexts of all three versions are the same, so that it makes no sense to deliver the same task more than once (the only difference being information format). The hospital problem, the Monty Hall problem, and the Linda problem were only presented in one version in general. Figure 1 displays the traditional versions implemented on the left and the facilitated versions on the right.

All traditional versions (Wason, AIDS, Linda, hospital problem) were only slightly modified in order to avoid guessing on the one hand and floor effects on the other. In the Wason task, for instance, we adjusted the wording (i.e., minor linguistic changes) of both well-known versions (i.e., the classic, context-free version with letters and numbers, and the contextualized version with stamped letters) in order to make the problem more easily understandable to students. Regarding the Bayesian reasoning task, we replaced the famous mammography context (which is usually not of relevance for 16-year-old students) by a context dealing with HIV tests. In addition, we added a tree diagram, which school students are familiar with (because in the probability version without a visualization, floor effects would be expected; Gigerenzer, 2004; Eichler and Vogel, 2015).

For the hospital problem, we changed the numerical values slightly and somewhat adapted the answer options to the question (students were instructed to check the boxes of three statements as to whether they were right or wrong). For the Linda task, in deviation from the traditional version, the students in our sample were asked to rank *three* available statements (instead of just naming the more probable statement out of two) and tick the boxes accordingly; this somewhat diminished the 50% probability of guessing the right answer.

However, we do not consider these changes systematic theoretical facilitations, which is why the Wason task (traditional), the hospital problem, and the Linda task are still displayed in Figure 1 on the left. In contrast, the reason for only presenting a facilitated version of the Monty Hall problem (right side of Figure 1) was that the original problem was simply too difficult and would probably yield floor effects (e.g., Krauss and Wang, 2003; Saenen et al., 2015). Instead, all three possible constellations (namely, where the main prize could be) were visualized according to Krauss and Atmaca (2004) and the cognitive illusion was further mitigated by specifying intermediate cognitive steps (e.g., in front of and to the right of the visualization) in which participants were explicitly asked for the number of constellations for which it would be worthwhile to change the door selection (i.e., thus triggering a frequency algorithm).

The order of the *cogIll* items in the questionnaire was as follows: First all four traditional (i.e., not facilitated) tasks were given, namely Wason classical, AIDS probability version (optional), hospital, and Linda, then the four simplified tasks, namely Monty Hall, Wason context, and AIDS frequency version 1 *or* 2 (if AIDS probability version was *not* provided). Since the implemented cognitive illusions, with the exception of the two Wason tasks (which were clearly separated from each other in the test booklet), differ substantially from each other in terms of mathematical structure and solution strategy, we refrained from randomizing the tasks for test economic reasons.

#### Mathematical Literacy (*ml*)

**FIGURE 2:**
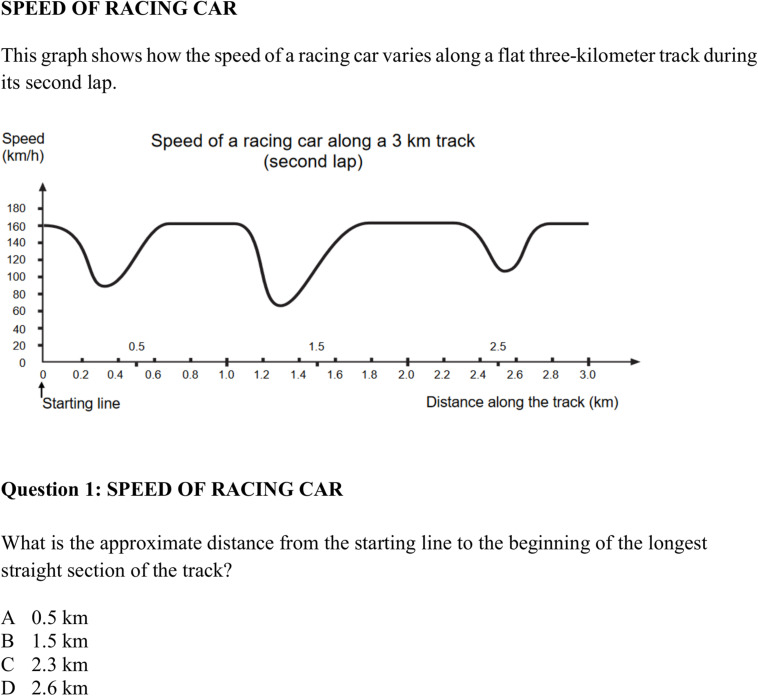
Mathematical task “Speed of Racing Car” [with one out of five questions; subscale “algebra”; from Organisation for Economic Co-operation and Development (OECD), 2003].

Mathematical competence was assessed using items from the mathematical literacy test (*ml*) originally implemented in PISA 2000 (Organisation for Economic Co-operation and Development (OECD), 2003). In more detail, *ml* was covered by items from the four areas of algebra (12 items), arithmetics (8 items), geometry (10 items), and stochastics (7 items) (see Figure 2 for a sample item). A complete compilation of all items covering *ml* can be found in the electronic Supplementary Material (ESM). For statistical analyses, four parcels (i.e., sum scores) of algebra, arithmetics, geometry, and stochastics form the manifest indicators for *ml*.

#### Intelligence (*g*)

**FIGURE 3:**
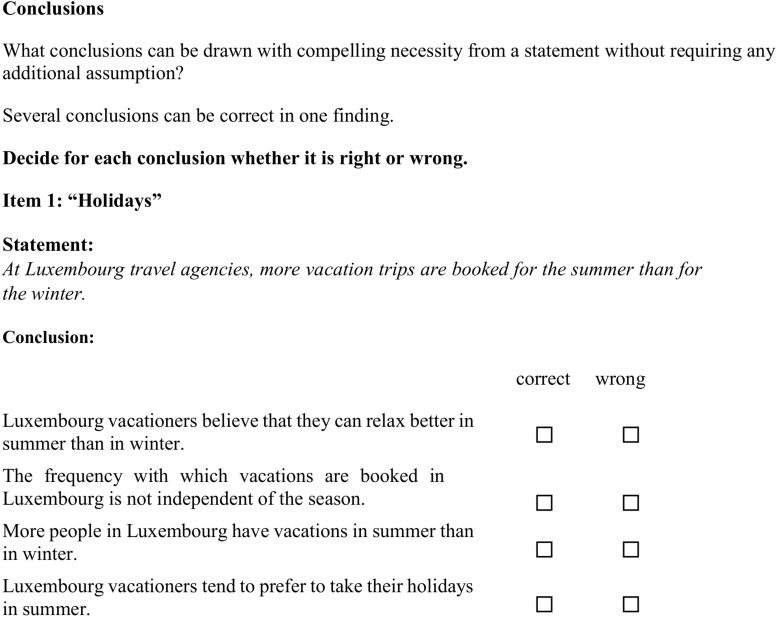
General intelligence items on the topic “Vacations” (from parcel 1; from Jäger et al., 1997).

To cover general intelligence (*g*), we implemented established reasoning items from the “Berliner Intelligence Structure test” (BIS; Jäger et al., 1997). Three different statements concerning different topics were provided (*Vacations*, *Traffic*, and *Smoking*; see Figure 3 for a sample statement). Then four possible conclusions were presented, each of which tested whether the statement was understood logically (i.e., there were four items per scenario). The three resulting sum scores regarding each of the three topics form the respective manifest parcels that were used as indicators for *g*. A complete compilation of all items covering *g* can be found in the ESM.

#### Reading Literacy (*rl*)

**FIGURE 4:**
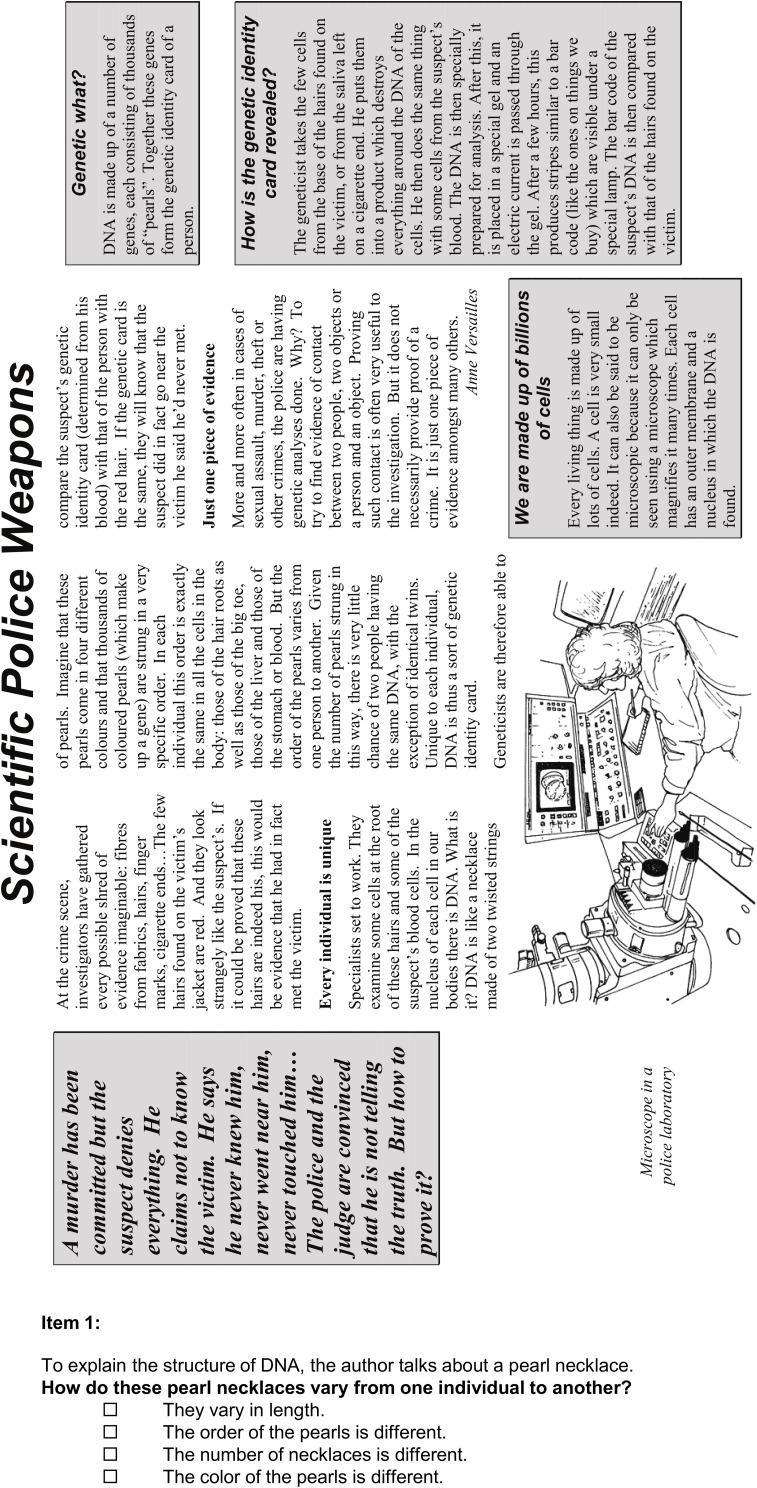
Reading task “Police” [with one out of 4 questions; from Organisation for Economic Co-operation and Development (OECD), 2003].

Since some of the brain teasers are formulated in a linguistically demanding way, reading literacy (*rl*) was also included in the present study. Four situations from the PISA 2000 reading test including a question and possible answer options in each scenario were implemented, resulting in 18 corresponding items altogether (for a sample scenario, see Figure 4). In more detail, *rl* was covered by items regarding the four descriptive texts *Lake Tchad* (three items), *Flu* (three items), *Labor* (eight items), and *Police* (four items). Unlike *g*, the items on *rl* require reading and in-depth comprehension of longer and more complex texts. A complete compilation of all items covering *rl* can be found in the ESM. For statistical analyses, four parcels (i.e., sum scores) of the items belonging to each of the four situations form the manifest indicators for *rl*.

#### Further Individual Covariates

In addition, further individual student characteristics were collected. They included sociodemographic background features (gender, age, etc.), learning motivation (e.g., interests and self-image), life goals, and life satisfaction.

### Participants

PROLOG was administered to Luxembourgian school students of age 16–18 in grades nine or ten who did not take part in the PISA study in 2009. In more detail, a total of 2,643 pupils (56% girls) from 19 different Luxembourgian schools participated in PROLOG. The average age M (SD) of the students was 16.31 (0.57) years. About half of the students completed the *Enseignement Secondaire Technique* (“EST”; the Luxembourgian non-academic vocational track), and the other half of the students the *Enseignement Secondaire* (“ES”; the Luxembourgian academic track required for university studies). 68% of the students attended grade nine (63% EST, 37% ES), while the other 32% attended grade ten (34% EST, 66% ES). Note that only the AIDS task (see section “Cognitive Illusions”), which was applied in three different versions, is an exception in terms of sample size. Each of the three AIDS versions was processed by only approximately 880 students of the total sample.

### Procedure

PROLOG took place in April and May 2009 during regular school hours. In the run-up to PROLOG, the research program was presented to all Luxembourgian secondary schools (i.e., the principals) in the form of a letter and the schools were encouraged to allow their students to participate. However, participation was not compulsory and remained optional for the schools on a voluntary basis.

In addition to the cognitive illusions and demographic questions, the PROLOG study included some scales of PISA 2000 and in total lasted about three and a quarter hours (test duration: 2 h 40 min, exclusive of an initial briefing of 15 min and two breaks, one 5 min and the other 15 min, during the test). The students were assured that the evaluation of the questionnaire would be anonymous and that the results of the study would in no way influence the grades of the individual student.

All measuring instruments were distributed in the form of one test booklet. PROLOG was conducted by teachers whose 15-year-old students were participating in PISA and who therefore were not teaching at the time. Those teachers distributed the test material, read out standardized instructions on how to fill in the PROLOG instruments, kept the students quiet during the test, and finally collected the PROLOG materials and handed it over to the PISA school coordinators for return.

### Statistical Analysis

While *ml*, *g*, and *rl* were treated as reflexive constructs based on manifest indicators (which in turn were parcels consisting of single items, see above), in the following, *cogIll* will be treated as a construct, but will also be considered at the individual item level.

All analyses were conducted using the open statistical software *R* (R Development Core Team, 2020). Regarding RQ 2, an unconditional random effects model (*UREM*) was used to estimate the between-task-type, between-participant, and between-school variances of the binary task results of the *cogIll* items, and to compare these three sources within class variance. Subsequently, to take nesting into account, the “lme4” package (Bates et al., 2015) and the “blme” package (Chung et al., 2013) were used to create separate frequentist and Bayesian generalized mixed regression models. More specifically, mixed logistic regressions were modeled, which used the following (logistic) link function to relate the linear term η to the probability of solving a task (meaning a result of *X* = 1):

$$P\left(X=1\right)=e^{\eta }/\left(1+e^{\eta }\right)$$

All models allowed for random intercepts, and the following indicators of model fit were estimated: *R*^2^_Marginal_ represents the variance explained by the fixed effects, and *R*^2^_Conditional_ represents the variance explained by both fixed and random effects as estimated using the “MuMIn” package (Barton, 2016).

Regarding RQ 2, four predictors were included in all models to predict outcomes concerning *cogIll*: *ml*, *g*, *rl*, and “task difficulty” *d* (i.e., facilitated or not), which was dummy-coded (*0: facilitated; 1: traditional version*).

A first model included these predictors in additive fashion within the linear term γ_*00*_ as intercept:

$$\eta =\gamma_{00}+m\,l+g+r\,l+d+M\,i\,x\,e\,d\,E\,r\,r\,o\,r\,T\,e\,r\,m\,s$$

Possible interaction effects between *d* (task difficulty) and the other three predictors were modeled via the inclusion of additional multiplication terms of the form *Predictor x Difficulty*. For a detailed description of the interpretation of such error terms with dummy-coded binary predictors in mixed models, see Hilbert et al. (2019). Type-I error probabilities for the significance of the regression estimates were corrected for sevenfold multiple testing according to *Bonferroni*, as a maximum of seven predictors was used for the models, meaning that *p* < 0.05/7 = 0.007 was regarded as statistically significant.

## Results

In the following, the results are presented according to the three research questions RQ 1a, RQ 1b, and RQ 2.

### Descriptives of *cogIll* and Reliability Analysis (RQ 1a)

All items of *cogIll* were coded dichotomously (0 = wrong; 1 = correct). Overall, the traditional versions (Figure 1, on the left) of the cognitive illusions, which were processed by *N* = 2,643 students, yielded expectedly low solution rates (Table 1). The four “original” items (i.e., without substantial facilitation) were only correctly solved by 8–16% of the students, specifically the Wason task (based on letters and numbers) by 14%, the hospital problem by 10%, the Linda task by 16%, and, finally, the AIDS task in probability format—despite the additional tree diagram—by only 2% (note that each of the three AIDS task versions was only handled by *N* ≈ 880 students). Regarding the “facilitated” versions (Figure 1, on the right), both natural frequency versions of the AIDS task were solved at a significantly higher rate (yet with solution rates still not over 10% or 11%). The facilitated Wason task (with the letter-stamp context) was solved by 29% and the Monty Hall problem, including various facilitations, by 67%.

**TABLE 1:** # correct solutions (in percent), standard deviations, and manifest intercorrelations of *cogIll* items including Cronbach’s alpha if item deleted.

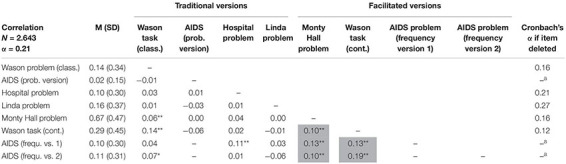

*indicates *p* < 0.05; **indicates *p* < 0.01. Correlations of facilitated items (cf. Figure 1) with each other are gray-shaded.

*The three AIDS versions as an exception were each only processed by ≈ 880 students.*

*^*a*^Cronbach’s α if item deleted of all versions of the AIDS task combined is 0.18.*

According to RQ 1a, the statistical analysis of the data showed a reliability of Cronbach’s α = 0.21 of *cogIll* (Table 1). The low value means that the individual brain teasers are only weakly related to each other, and there seems to be no distinguished general ability to “see through cognitive illusions.” Although the internal consistency could be increased up to an alpha of 0.27 by, for instance, deleting the Linda task, there is no way to arrive at the satisfying reliability level usually requested for reflexive constructs (e.g., Bühner and Ziegler, 2017). However, keep in mind that the chosen famous brain teasers cover different contents, require cognitively varying solution strategies, and tempt to different traps.

Table 1 (in which all items are listed according to the administration order) shows that the correlations between the *cogIll* items are at a very low level and in some cases even show—at least descriptively—negative values. The significant, but small correlation effect between the two versions of the Wason task of *r* = 0.14 indicates that it was reasonable to implement both tasks simultaneously (note that due to the large sample size, small correlations can also become significant). No mutual intercorrelations between the three AIDS task variants can be obtained because each participant only had to solve one of them (also see legend of Table 1).

A closer inspection of Table 1 reveals a remarkable result: *Facilitated* items show substantial correlations to each other. Separating both problem modes yields the corresponding reliabilities α*_*cogIll*__*orig.*_* = −0.01 compared to α*_*cogIll facilit.*_* = 0.30. Thus, interestingly, while the original problems indeed seem to be solved only randomly, the facilitations are what make the problems somehow accessible to consistent cognitive processing. This result is strengthened by the fact that while the natural frequency versions of the AIDS task display substantial correlations to other facilitated items, the corresponding AIDS probability version does not.

### Relationship and Confirmatory Factor Analysis of *cogIll*, *ml*, and *g* (RQ 1b)

In order to address the relationship between *cogIll*, *ml*, *g*, and *rl*, we first present the descriptive results on the four constructs, including their manifest mutual intercorrelations. Although it will not be part of the confirmatory factor analysis, we include *rl* here because it will be used later as an additional predictor in the regression analyses with respect to RQ 2. Student performance regarding the three constructs *ml*, *g*, and *rl* (see Table 2) lies, as expected and in contrast to *cogIll*, at an average level (i.e., students solved about half of the items concerning all three abilities). The internal consistencies were—except for intelligence *g*—satisfactory (and all clearly above the reliability of *cogIll*). However, α_g_ = 0.43 for *g* also corresponds to an acceptable value given the fact that it is a rather broad scale including three completely different scenarios and statements. As is abundantly clear from many PISA cycles, *ml* and *rl* are strongly correlated (*r* = 0.69), and each is also correlated with *g*, though less strongly (Table 2).

**TABLE 2:** Descriptives (M, SD, α) of and mutual (manifest) intercorrelations r (according to Spearman) between the constructs *cogIll*, *ml*, *g*, and *rl.*

***Competence***	**Theory max.**	**M (SD)**	**α**	***cogIll***	***ml***	***g***	***rl***
*cogIll*	6	1.41 (0.99)	0.21	–			
Mathematical literacy (*ml*)	34	14.16 (6.10)	0.82	0.42**	–		
General intelligence (*g*)	12	4.82 (1.73)	0.43	0.16**	0.25**	–	
Reading literacy (*rl*)	14	7.01 (3.17)	0.74	0.36**	0.69**	0.23**	–

**indicates *p* < 0.01.

Most importantly, despite the low internal consistency of *cogIll*, taken as a construct it displays significant (manifest) correlations with the other three constructs (the highest with *ml*, the lowest with *g*). Interestingly, *ml* and *rl* relate approximately equally to *cogIll*. However, since *cogIll* is not a homogeneous scale (cf. RQ 1a), correlations with *cogIll* cannot be generalized to individual tasks (see also next paragraph; Cohen, 1992). Considering the small reliability of *cogIll* (α*_*cogIll*_* = 0.21), it is rather informative to consider in addition the differential relationships of *ml*, *g*, and *rl* to each individual item of *cogIll*.

Regarding Table 3, the following three results are interesting: First, each single item of *cogIll* correlates very similarly with *ml* and with *rl* (only the hospital problem clearly depends more on *ml* than on *rl*). Second, for most (but not all) items of *cogIll*, the correlation with *g* lies below the correlations with *ml* and *rl* (which can only partly be explained with the medium reliability of *g*). And third (and most importantly), the facilitated versions correlate more strongly not only with each other (RQ1a), but also with the three constructs *ml*, *g*, and *rl*.

**TABLE 3:** Correlations of individual items from *cogIll* with *ml*, *g*, and *rl.*

**Correlations**	**Traditional versions**				**Facilitated versions**			
**Task *Competence***	**Wason (class.)**	**AIDS prob. vs.**	**Hospital problem**	**Linda problem**	**Wason (cont.)**	**AIDS frequ. vs. 1**	**AIDS frequ. vs. 2**	**Monty Hall**
*ml*	0.11**	0.01	0.09**	−0.01	0.30**	0.22**	0.21**	0.33**
*g*	0.03	−0.02	0.05*	0.03	0.11**	0.09**	0.13**	0.12**
*rl*	0.08**	−0.03	0.00	−0.05*	0.27**	0.18**	0.20**	0.32**

*indicates *p* < 0.05; **indicates *p* < 0.01.

This third—and most intriguing—result means that mathematical and reading skills (and also, to a lesser extent, intelligence) can only help when cognitive illusions are simplified with didactic measures and thereby made more accessible to those abilities. Regarding the *cogIll* items presented in their traditional versions, there are weaker and mostly not significant correlations throughout (*r* = −0.05–0.11), meaning that neither *ml* nor *g* nor *rl* can be effective here. This is in line with the provocative statements from Piattelli-Palmarini (1991) and Gould (1992; see section “Person-Related and Task-Related Characteristics Associated With the Ability to Solve Cognitive Illusions”) but contradicts, for instance, the threshold hypothesis regarding numeracy (Hill and Brase, 2012) and related findings from Stanovich (2012), who reported correlations between probabilistic reasoning abilities (even though not specifically concerning cognitive illusions) and cognitive ability (*g*) to be roughly in the range of 0.20–0.35.

**FIGURE 5:**
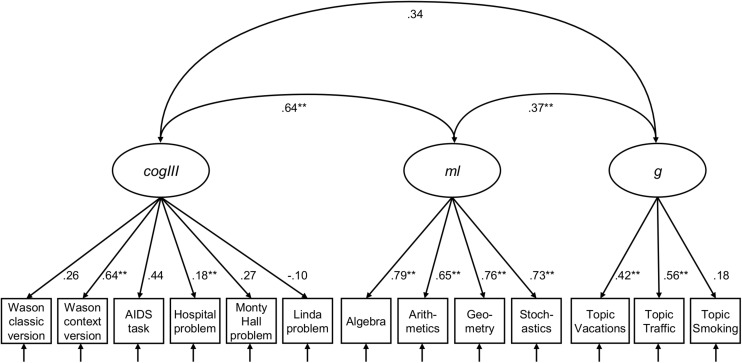
Three-factor measurement model of *cogIll*, *ml*, and *g.* Model fit: *N* = 2,508, *T* [χ*^2^*] = 103.796, *df* = 51, *p* = 0.001, CFI = 0.990, *TLI* = 0.987, *RMSEA* = 0.017, *SRMR* = 0.084. The values display latent correlation or standardized coefficients, respectively. Values of χ*^2^* ≤ 3*df* (*df* = degrees of freedom), *p* ≥ 0.01, CFI (Comparative Fit Index) ≥ 0.95, TLI (Tucker-Lewis Index) ≥ 0.95, RMSEA (Root-Mean-Square Error of Approximation) ≤ 0.05, and SRMR (Standardized Root Mean Residual) ≤ 0.05 indicate a good model fit. *cogIll*: cognitive illusions, *ml*: mathematical literacy, *g*: general intelligence. *indicates *p* < 0.05; **indicates *p* < 0.01.

With these results in mind, we now turn to the inspection of the dimensionality of *cogIll*, *ml*, and *g* with a latent confirmatory factor analysis (CFA, RQ1b). Note that *rl* was only intended as a moderator in the study, since according to the literature, statistical and logical reasoning is much more closely related to intelligence and mathematics abilities (thus *rl* was not of theoretical interest with respect to a common model^2^). The three included constructs (Figure 5) were formed from the manifest values of the six single items of *cogIll* (Wason classic, Wason context, Monty Hall problem, AIDS task, hospital problem, and Linda task), the four facets of *ml* (parcels: algebra, arithmetics, geometry, and stochastics) and the three facets of *g* (parcels: Vacations, Traffic, and Smoking). The CFA revealed adequate local and global fit [χ^2^(2,508, 51) = 103.796, *p* = 0.001, *CFI* = 0.990, *TLI* = 0.987, *RMSEA* = 0.017, *SRMR* = 0.084].

**FIGURE A1:**
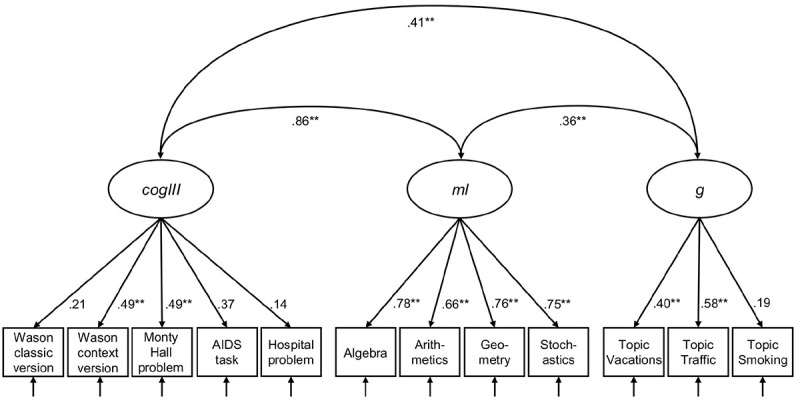
Three-factor measurement model of *cogIll*, *ml*, and *g.* Model fit: *N* = 2,508, *T* [χ*^2^*] = 120.786, *df* = 51, *p* = 0.000, *CFI* = 0.986, *TLI* = 0.982, *RMSEA* = 0.023, *SRMR* = 0.036. The values display latent correlation or standardized coefficients, respectively. Values of χ*^2^* ≤ 3*df* (*df* = degrees of freedom), *p* ≥ 0.01, CFI (Comparative Fit Index) ≥ 0.95, TLI (Tucker-Lewis Index) ≥ 0.95, RMSEA (Root-Mean-Square Error of Approximation) ≤ 0.05, and SRMR (Standardized Root Mean Residual) ≤ 0.05 indicate a good model fit. *cogIll*: cognitive illusions, *ml*: mathematical literacy, *g*: general intelligence. *indicates *p* < 0.05; **indicates *p* < 0.01.

As can be seen in Figure 5, *cogIll* and *ml* display a strong latent correlation (*r* = 0.64), while the other two latent correlations are substantially lower. The magnitude of the individual factor loadings of *cogIll* illustrate that again it is mainly the simplified tasks that contribute to the construct, while the loadings for *ml* and *g* are consistently high or moderate, respectively. Note that the fit indices remain pretty much the same if the Linda task were excluded from the model [model fit: *N* = 2,508, *T* (χ*^2^*) = 120.786, *df* = 51, *p* = 0.000, *CFI* = 0.986, *TLI* = 0.982, *RMSEA* = 0.023, *SRMR* = 0.036; see Appendix Figure A1].

### Predicting the Ability to Solve Brain Teasers (RQ 2)

Finally, we will predict the solution of the brain teasers of *cogIll*—each as a construct and individually—by means of regression models. In contrast to the correlational analyses (section “Relationship and Confirmatory Factor Analysis of *cogIll*, *ml*, and *g*”), the modeled predictors can now statistically control for each other.

#### Preliminary Models

First, an unconditional random effects model (UREM) was estimated to compare the degrees of variance of the three nesting levels (task difficulty, participant, and school). The highest variance accounted for was difficulty *d* (σ^2^ = 1.23), followed by participant-specific differences (σ^2^ = 0.18) and differences between the schools (σ^2^ = 0.09), with *R*^2^*_*Conditional*_* = 0.38. This means that the most significant factor explaining differences in performance regarding cognitive illusions relates to the “facilitation factor” *d* (separating between traditional and facilitated versions), which is why we include this dummy variable into the following models in addition to *ml*, *g*, and *rl*.

#### Direct Effect of Change Factors on *cogIll*

We then investigated the direct influence of *ml*, *g*, *rl*, and *d* on the solution of cognitive illusions using several models (see Table 4). Because standard frequentist regression models showed convergence problems, the standard optimizer was exchanged for the “bobyqa” optimizer, and the convergence tolerance was set to 0.01. These convergence problems usually stem from multicollinearity and are likely to be due to the strong correlation of the covariates *ml* and *rl* (see Table 2). To double-check the results obtained from these models, additional Bayesian mixed regression models with Wishart priors for the covariance distributions were estimated, using the same sets of predictor variables. As can be seen in Table 4 and Appendix Table A1 (where the corresponding Bayesian models can be found), both types of regression models show identical patterns of significant predictors for performance in *cogIll*.

**TABLE 4:** Predictiveness of different factors (i.e., *ml*, *g*, *rl*, and *d*) regarding the criterion *cogIll* in two different frequentist models (with and without interactions).

**Frequentist Model**	***Estimate***	***SE***	***p***	***Explained variance***
Without Interactions				*R*^2^*_Marginal_* = 0.054; *R*^2^*_*Conditional*_* = 0.040
γ_00_	−**2.2**	**0**.**575**	**< 0.001**	
*ml*	**0.067**	**0**.**006**	**< 0.001**	
*g*	**0.039**	**0**.**012**	**0.001**	
*rl*	−**0.028**	**0**.**014**	**0.04**	
*d*	–1.074	0.804	0.182	
With Interactions				*R*^2^*_Marginal_* = 0.107; *R*^2^*_*Conditional*_* = 0.348
γ_00_	**−3.039**	**0.615**	**< 0.001**	
*ml*	**0.087**	**0.008**	**< 0.001**	
*g*	**0.046**	**0.016**	**0.005**	
*rl*	0.022	0.018	0.234	
*d*	0.822	0.867	0.343	
*ml* × *d*	−**0.042**	**0**.**012**	**< 0.001**	
*g* × *d*	–0.015	0.024	0.536	
*rl* × *d*	–0.124	0.027	**< 0.001**	

*Model fit: CFI: 0.990, RMSEA: 0.017.*

*Estimate, Estimated unstandardized parameter value; SE, Standard error of the parameter estimate; *df*, Degrees of freedom; *p*, Probability of committing a Type I Error; γ_00_, Intercept of the additive predictor term; *R*^2^_*Marginal*_, Variance explained by fixed effects; *R*^2^_*Conditional*_, Variance explained by both fixed and random effects.*

*Significant (direct or interaction) effects (*p* < 0.05) are written in bold. Corresponding Bayesian models as well as models without the Linda task can be found in Appendix Tables A1, A2.*

The results in Table 4 show three significant factors of influence for *cogIll*: specifically, the models using only the additive linear term (i.e., without interaction effects) show that *ml*, *rl*, and *g* significantly predicted the probability of solving a cognitive illusion, while the item difficulty *d* interestingly showed no significant impact. Additionally, the models including the interaction terms showed a significant negative interaction effect of both *ml x d* and *rl x d* (whereas also due to the interaction effect of *rl x d*, the direct effect of *rl* is no longer predictive). This means that higher mathematical and reading skills were associated with less of an influence of task difficulty or, in other words, the facilitating measures taken to help the participants to solve the brain teasers were more helpful for (or needed by) those students with lower mathematical and reading skills.

To check the possible influence of the exclusion or inclusion of the Linda task, we also calculated the identical regression models (i.e., with and without interaction terms) without the Linda task (cf. Appendix Table A2). In the linear model, both *ml* and *g* (but not *rl*) were significant predictors of the probability of solving cognitive illusions. In the model with interaction terms, all effects except for the interaction effect *ml x d*, which was no longer predictive, remained the same compared to the models including the Linda task.

Because of the low correlations of the cognitive illusions with each other (see Table 1), it is reasonable to consider the prediction of solving the *individual* brain teasers in addition. Corresponding regression models (not depicted in Table 4) revealed differential regression coefficients, especially regarding reading literacy *rl*. While *rl* had almost no effect on, for instance, performance on the hospital task, it was a relatively strong predictor on text-intensive or context-rich problem formulations like the Monty Hall problem or the Wason selection task (with the letter-stamp context).

## Discussion

In this paper we inspect famous statistical and logical cognitive illusions from the heuristics and biases research program of Daniel Kahneman and Amos Tversky from a psychometrical perspective. With a sample of *N* = 2,643 Luxembourgian students of age 16 to 18, we implemented the Wason card selection task (on the understanding of logical implication and its reversion), the hospital problem (on the empirical law of large numbers), the Linda task (on the conjunction rule for multiplying probabilities), the AIDS task (a Bayesian reasoning problem analogous to the famous mammography task), and the Monty Hall problem (a special case of a Bayesian reasoning problem, which was not part of the heuristics and biases program by Kahneman and Tversky).

Over the last few decades, many researchers (especially from the research group of the German psychologist Gerd Gigerenzer) have made attempts to modify information representation and in that way make these kinds of brain teasers more accessible to human thinking processes. These variations were acknowledged as an experimental factor, meaning that some of the brain teasers were implemented in a version very close to their original formulations (e.g., the Linda and the hospital problem), and some in a facilitated way, in order to avoid both guessing and floor effects (e.g., the Monty Hall problem). Because the contexts of the classical Wason task (based on numbers and letters) and the corresponding facilitated version (based on stamps and letters) substantially differ in the present study, it was possible to implement both versions simultaneously for all participants. Regarding the Bayesian AIDS task, a traditional version (based on probability format) and two facilitated versions (based on frequency format) were implemented, yet (in contrast to the Wason task) only one of these versions was presented to each participant.

So far, these cognitive illusions have been described together within the theoretical framework of the heuristics and biases program (and explained, e.g., by representativeness or confirmation bias) or the more comprehensive framework CART. Yet, experiments astoundingly have usually only implemented one of these brain teasers empirically at the same time. Explicitly addressing this research desideratum, our design included all mentioned illusions simultaneously.

Based on our sample of Luxembourgian students of age 16–18, we found that these brain teasers were only moderately correlated to each other, yielding a low reliability of an assumed reflexive construct *cogIll* (α = 0.21, or a maximum of 0.27 without the Linda task). Interestingly, this (small) amount of shared variance was exclusively due to the facilitated versions, while the reliability of the remaining traditional versions was almost zero. Analyses of manifest correlations revealed that *cogIll* was substantially correlated to intelligence (*g*) and mathematical and reading competence (the correlations to the two latter ones, *ml* and *rl*, which were operationalized by parts of the corresponding PISA tests, were even higher than for *g*). On the individual item level, these correlations were again much higher for the facilitated versions, giving a first hint that the above-mentioned literacies (*ml* and *rl*) and the general cognitive ability (*g*) cannot be applied properly to the traditional versions. In a subsequent confirmatory factor analysis (where *rl* was excluded because of multicollinearity), a latent construct *cogIll* could be modeled and distinguished from *g* and *ml*, yet still displaying a high latent correlation to *ml*.

Finally, we ran a series of frequentist and Bayesian regression models (both with and without interaction terms) in order to predict the correct solving of the brain teasers both on construct and on individual item level. The best predictor across all implemented models was mathematical competence, followed by intelligence. Interestingly, the (negative) interaction effect of *rl* x *d* (with *d* being the dummy variable indicating whether the problem representation was facilitated or not) suggests that the systematic facilitating measures taken to help the participants to solve the brain teasers were more helpful for (or needed by) those students with lower reading skills. Since the original versions of the cognitive illusions obviously make it very difficult to extract the relevant information and then to infer the correct answer, it seems that these traditional formulations (and not the tasks or the underlying mathematical structure *per se*) in a way trigger cognitive bias. Thus “facilitation” is about translating information into a more accessible form, which partially “disarms the trap” and thus makes it easier for people to apply their general or content-specific skills to the tasks. Furthermore, considering the individual item level of *cogIll*, reading literacy was particularly necessary for text-intensive and context-rich problems such as the Monty Hall problem.

Of course, the present study can only shed a first light on psychometric properties of the brain teasers, on their mutual correlations, and on connections to related constructs. Empirically examining some of these brain teasers together, however, the study goes beyond comprehensive but more theoretical compilations of reasoning items (cf. CART; Stanovich, 2016). Future studies could (1) implement further cognitive illusions of the heuristics and biases program, (2) vary the facilitation manipulation more systematically, (3) use additional constructs for both confirmatory factor and regression analyses, or (4) administer similar studies with adult samples. However, we hope to have opened a path toward the consideration and empirical investigation of statistical and logical cognitive illusions not only at an individual item level, but also at the level of a psychometric construct.

## Data Availability Statement

There is no public use file available for the data used in the present study. Requests to access the present data should be directed to MB, martin.brunner@uni-potsdam.de.

## Ethics Statement

Ethical review and approval was not required for the study on human participants in accordance with the local legislation and institutional requirements. Written informed consent from the participants’ legal guardian/next of kin was not required to participate in this study in accordance with the national legislation and the institutional requirements.

## Author Contributions

GB, SK, and KB contributed by writing the draft of the manuscript while SH conducted the data analysis. MB planned and conducted the study. All authors listed have made a substantial, direct and intellectual contribution to the work, and approved it for publication.

## Conflict of Interest

The authors declare that the research was conducted in the absence of any commercial or financial relationships that could be construed as a potential conflict of interest.

## Footnotes

1 In 2002, Daniel Kahneman was indeed awarded with the Nobel prize in economics.

2 Furthermore, including *rl* in the latent CFA would also lead to convergence problems due to its strong correlation with *ml*.

## Appendix

**TABLE A1:** Predictiveness of different factors (i.e., *ml*, *g*, *rl*, and *d*) regarding the criterion *cogIll* in two different Bayesian models (with and without interactions) *including* the Linda task.

**Bayesian Model**	***Estimate***	***SE***	***p***	***Explained variance***
**Without Interactions**				/
γ_00_	−**2.205**	**0.664**	**0.001**	
*ml*	**0.067**	**0.006**	**<0.001**	
*g*	**0.039**	**0.012**	**0.002**	
*rl*	−**0.028**	**0.014**	**0.042**	
*d*	–1.077	0.93	0.247	
**With Interactions**				/
γ_00_	**−3.045**	**0.71**	**<0.001**	
*ml*	**0.087**	**0.008**	**<0.001**	
*g*	**0.046**	**0.016**	**0.005**	
*rl*	0.022	0.018	0.23	
*d*	0.825	1.002	0.411	
*ml* × *d*	−**0.042**	**0.012**	**<0.001**	
*g* × *d*	–0.015	0.024	0.534	
*rl* × *d*	–0.124	0.027	**<0.001**	

*Estimate, Estimated unstandardized parameter value; SE, Standard error of the parameter estimate; *df*, Degrees of freedom; *p*, Probability of committing a Type I Error; γ_00_, Intercept of the additive predictor term; *R*^2^_*Marginal*_, Variance explained by fixed effects; *R*^2^_*Conditional*_, Variance explained by both fixed and random effects. Significant (direct or interaction) effects (*p* < 0.05) are written in bold.*

**TABLE A2:** Predictiveness of different factors (i.e., *ml*, *g*, *rl*, and *d*) regarding the criterion *cogIll* in four different models (frequentist and Bayesian, both with and without interactions) *without* the Linda task.

**Frequentist Model**	***Estimate***	***SE***	***p***	***Explained variance***
**Without Interactions**				*R*^2^*_Marginal_* = 0.131; *R*^2^*_Conditional_* = 0.391
γ_00_	−**2.533**	**0.65**	−**3.9**	
*ml*	**0.078**	**0.007**	**11.53**	
*g*	**0.038**	**0.014**	**2.767**	
*rl*	–0.016	0.015	–1.026	
*d*	–1.252	1.014	–1.234	
**With Interactions**				*R*^2^*_Marginal_* = 0.123; *R*^2^*_Conditional_* = 0.403
γ_00_	−**3.047**	**0.679**	−**4.486**	
*ml*	**0.086**	**0.008**	**1.489**	
*g*	**0.046**	**0.017**	**2.786**	
*rl*	0.022	0.019	1.182	
*d*	0.325	1.07	0.304	
*ml* × *d*	–0.02	0.013	–1.508	
*g* × *d*	–0.023	0.028	–0.815	
*rl* × *d*	−**0.13**	**0.031**	−**4.141**	
***Bayesian Model***				
**Without Interactions**				/
γ_00_	−**2.539**	**0.773**	−**3.285**	
*ml*	**0.078**	**0.007**	**11.365**	
*g*	**0.038**	**0.014**	**2.735**	
*rl*	–0.016	0.016	–1.004	
*d*	–1.255	1.206	–1.04	
**With Interactions**				/
γ_00_	−**3.054**	**0.81**	−**3.769**	
*ml*	**0.086**	**0.008**	**1.451**	
*g*	**0.046**	**0.017**	**2.783**	
*rl*	0.022	0.019	1.188	
*d*	0.328	1.28	0.256	
*ml* × *d*	–0.021	0.013	–1.521	
*g* × *d*	–0.023	0.028	–0.82	
*rl* × *d*	−**0.13**	**0.031**	−**4.146**	

*Model Fit (Frequentist Model): CFI: 0.990, RMSEA: 0.017.*

*Estimate, Estimated parameter value; SE, Standard error of the parameter estimate; *df*, Degrees of freedom; *z, z*-value; *p*, Probability of committing a Type I Error; γ_00_, Intercept of the additive predictor term; *R*^2^_*Marginal*_, Variance explained by fixed effects; *R*^2^_*Conditional*_, Variance explained by both fixed and random effects.*

*Significant (direct or interaction) effects (*p* < 0.05) are written in bold.*
