# Supplementary material for: Tversky and Kahneman’s Cognitive Illusions: Who Can Solve Them, and Why?
Source: Front Psychol. 2021 Apr 12;12:584689. doi: 10.3389/fpsyg.2021.584689 (PMC8075297; doi:10.3389/fpsyg.2021.584689)
Supplement: Supplementary file 1 [file Data_Sheet_1.docx]

Supplementary Material

# Mathematical literacy (*ml*)

In a similar way to the reading tasks, PROLOG-1 also used a few published PISA tasks (from 2006) to assess mathematical skills. In more detail, 30 tasks with several (sub)items each were implemented. These tasks with the corresponding questions are depicted in the following. For a full list of all published PISA 2006 items covering mathematical literacy (incl. the corresponding scoring), also see https://www.oecd.org/pisa/38709418.pdf.

## Algebra (12 items)

### Algebra task 1: “Speed of a Racing Car” (3 items)

**Speed of a Racing Car**

This graph shows how the speed of a racing car varies during its second lap on a three‑kilometer long flat race track.

Speed of a racing car on a distance of
3 km (2nd lap)


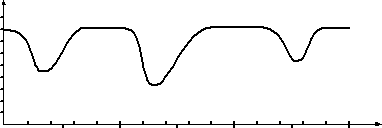


0.5 1.5 2.5

Speed

(km/h)

Distance (km)

180

160

140

120

100

80

60

40

20

0

0 0.2 0.4 0.6 0.8 1.0 1.2 1.4 1.6 1.8 2.0 2.2 2.4 2.6 2.8 3.0

Starting line

**Task 1: Racing Car**

**What is the approximate distance from the start line to the start of the longest straight section of the circuit?**

0.5 km

1.5 km

2.3 km

2.6 km

**Task 2: Racing Car**

**Where was the lowest speed measured during the second lap?**

at the start line

at about 0.8 km

at about 1.3 km

at the half-way mark

**Task 3: Racing Car**

**What can you say about the speed of the car between the 2.6 km and 2.8 km markers?**

The speed of the car remains constant.

The speed of the car is increasing.

The speed of the car is decreasing.

The speed of the car cannot be determined from the graph.

### Algebra task 2: “Two numbers” (3 items)

**Two numbers**

**Task 1**

For two numbers x and y shall apply x + y = 1.

**Mark the correct statement with a cross.**

If x is negative, then y is also negative.

If x is greater than 1, then y is also greater than 1.

Neither x nor y can be negative.

If x is smaller than 1, then y is positive.

x and y must have different signs.

**Task 2**

For two numbers x and y shall apply x ⋅ y = 1.

**Mark the correct statement with a cross.**

If x is negative, then y is positive.

If x is greater than 1, then y is also greater than 1.

Neither x nor y can be negative.

If x is smaller than 1, then y is negative.

x and y must have the same sign.

**Task 3**

For two numbers x and y (y ≠ 0) shall apply $\frac{\text{x}}{\text{y}}$ = 1.

**Mark the correct statement with a cross.**

If x is negative, then y is positive.

If x is greater than 1, then y is also greater than 1.

Neither x nor y can be negative.

If x is smaller than 1, then y is negative.

x and y must have different signs.

### Algebra task 3: “Mathematics and reality” (3x4 items)

**Mathematics and reality**

**Which statement fits the given equation?**

**Task 1:**

**y = 1.25 x** right wrong

There's 1.25 m of snow.

The time it takes a jogger to cover 1.25 km depends on his speed.

Due to increased security costs, air tickets are increased by 1.25 €.

When filling up, you pay 1.25 € per liter of premium petrol.

**Task 2:**

**y = - 2x + 30** right wrong

A tunnel is 30 m long. Every day, 2 m are dug anew.

With an account balance of -2 €, 30 € will be paid in.

A candle has a height of 30 cm. Every hour 2 cm burn off.

In a class 2 € are collected from each student. Of this amount 30 € will be donated.

**Task 3:**

| **y = x + (x + 2) = 40** | right | wrong |
| --- | --- | --- |
| Uwe has 40 blank CDs from two different manufacturers. He has twice as many of one kind as of the other. |  |  |
| Hans is 2 years older than Klaus. Together they are 40 years old. |  |  |
| Anna and Lisa cover a total of 40 km on their two-day hike. On the second day they cover 2 km more than on the first day. |  |  |
| Gabi is 40 years old. Her daughter Nina is 2 years older than her son Stefan. |  |  |

### Algebra task 4: “Points” (3 items)

**Points**

The following equation is given: y = 2x^2^ + 3x - 1

**Are the points** **P (x | y) on the graph of the function?**

P (x | y) yes no

P (2 | 13)

P (0 | 4)

P (5 | -1)

### Algebra task 5: “Graphs” (6 items)

**Graphs**

Graphs of functions are sketched here:

**Which graph belongs to the functions in the table?**

Write the letter of the graph belonging to the function into the table below.

| **A** | y  x | **B** | 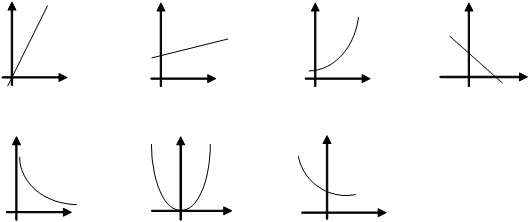  y  x | **C** | y  x | **D** | y  x |
| --- | --- | --- | --- | --- | --- | --- | --- |
| **E** | y  x | **F** | y  x | **G** | y  x |  |  |

**function belongs to graph**

**A B C D E F G**

y = 0.5x + 2

y = 2x

y = 2 - x

y = x^2^

y = $\frac{\text{1}}{\text{x}}$

y = 2^x^

### Algebra task 6: “Bacteria”

**Bacteria**

Under favorable conditions, bacteria multiply quite rapidly. Assuming the number of bacteria doubles every 2 hours:

**How many bacteria can be produced from a single bacterium in a day?**

12

24

512

1,024

4,096

## Arithmetics (8 items)

### Arithmetics task 1: “Market garden”

**Market garden**

In a market gardening business, you can expect to make big losses.

The gardener needs 600 plants.

**How many plants must be planted if only 60% of all plants survive?**

240

360

840

960

1,000

### Arithmetics task 2: “Numbers”

**Numbers**

**To which number do you have to add 6,345 to get 8,567?**

Mark the correct answer with a cross.

2,023

2,222

1,987

14,912

### Arithmetics task 3: “Sum of the digits”

**Sum of the digits**

The sum of the digits of a number is obtained by adding up its digits.

Example: The number 3,104 has the sum of 3 + 1 + 0 + 4 = 9.

**What is the smallest four-digit number with the sum of the digits of 12?**

Mark the correct answer with a cross.

129

1,002

1,029

1,119

1,236

### Arithmetics task 4: “Glass factory”

**Glass factory**

A glass factory produces 8,000 bottles a day. Experience shows that about 160 bottles are faulty.

**What percentage is that?**

Mark the correct answer with a cross.

0.02 %

0.5 %

1.28 %

2 %

5 %

### Arithmetics task 5: “Petrol pump”

**Petrol pump**

A petrol station in Germany informs with the sticker “73 cent tax per euro” about the tax burden on the price of petrol.

At a price per liter of 123.9 cents/liter, a motorist filled up a total of 47.22 liters for 58.51 €.

**How much does the German state receive in taxes?**

Mark the correct answer with a cross.

15.80 €

34.47 €

42.71 €

73.00 €

90.45 €

### Arithmetics task 6: “Discount” (2 items)

**Discount**

Electric-Fischbach wants to expand its sales assortment. In the future, the store wants to be able to offer MP3 players with improved storage capacity.

Fischbach receives the following offer from the manufacturing company:

The purchase price for an MP3 player is 40 €. If at least 50 units are purchased, this price will be reduced by 5%, if at least 100 units are purchased, 10% and if at least 150 units are purchased, 15% discount will be given.

**Mark each statement with a cross, whether it is right or wrong**.

right wrong

If Electric-Fischbach buys 35 pieces, he gets a discount of 140 € in total.

If Electric-Fischbach buys at least 50 but at most 75 pieces, he gets a discount of 2 € per piece.

### Arithmetics task 7: “Rounding”

**Rounding**

Two different natural numbers are rounded to tens. In both cases you get 20.

**By how many ones can the two numbers differ at most?**

Mark the correct answer with a cross.

By 3 ones

By 4 ones

By 5 ones

By 9 ones

By 10 ones

### Arithmetics task 8: “Classes neiges”

**Classes neiges**

In the “Classes neiges” the students are divided into snowboard and ski courses. The number of students in the snowboard courses is 3:5 in relation to the number of students in the ski courses. 32 students in total take part in the “Classes neiges”.

**How many students are in the snowboard courses?**

Mark the correct answer with a cross.

8

12

19

20

27

## Geometry (10 items)

### Geometry task 1: “Cornflakes”

**Cornflakes**

**Are the following statements right or wrong?**


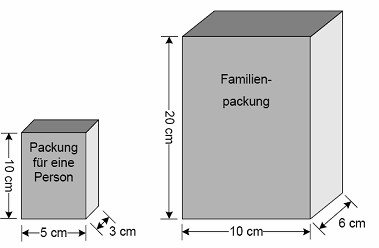


Family Pack

Pack for one person

The two packs for cornflakes shown have the same shape and are both completely filled with cornflakes. The small pack contains the amount of cornflakes that is normally enough for one person.

**How many such portions of cornflakes does the family pack contain?**

2 portions

4 portions

6 portions

8 portions

12 portions

### Geometry task 2: “Speed of a racing car” (part II, 1 item)

**Speed of a racing car**

This graph shows how the speed of a racing car varies during its second lap on a three‑kilometer long flat racetrack.


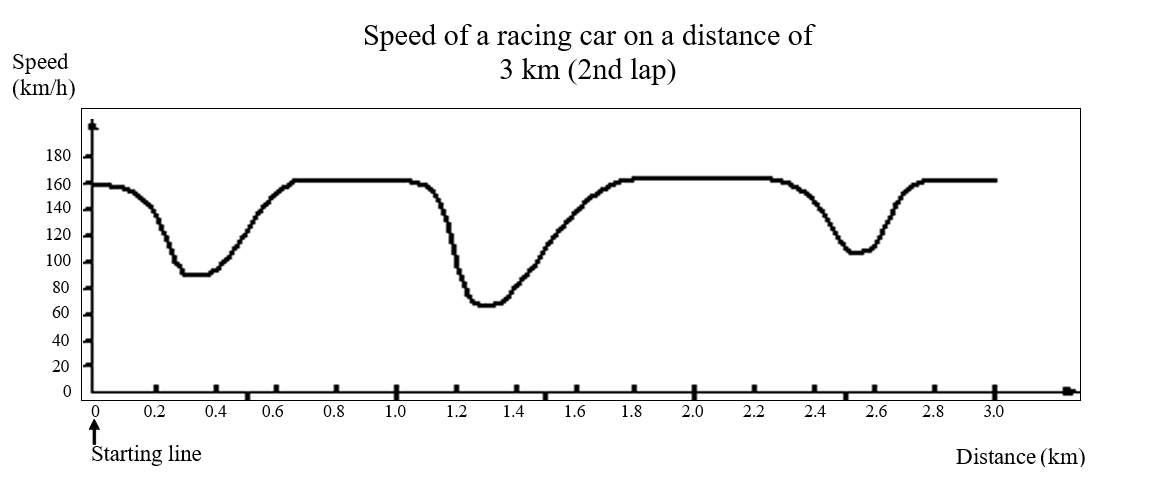
**Task 4: Racing Car**

Here you see pictures of five racetracks:


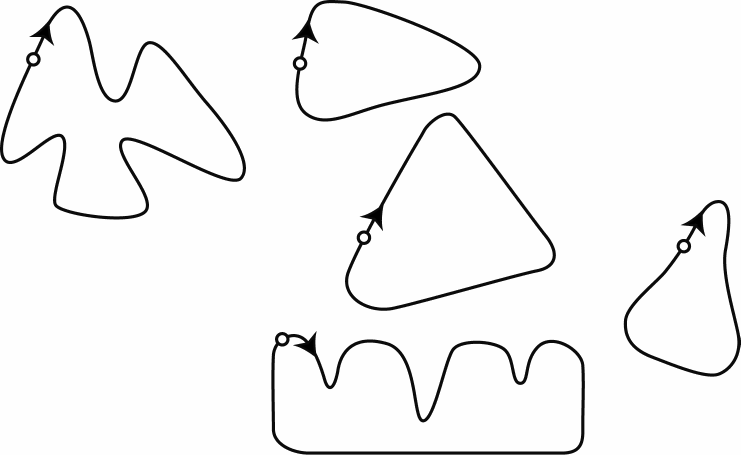


**S**

**S**

B

A

**S**

C

**S**

D

**S**

E

S: Starting line

**On which of these racetracks was the car driving, so that the speed graph shown at the beginning was created?**

racetrack A

racetrack B

racetrack C

racetrack D

racetrack E

### Geometry task 3: “Rectangle”

**Rectangle**

A rectangle is 4 cm long and 3 cm wide.

4 cm

3 cm

(drawing not dimensionally accurate)

**How large is its surface area?**

Mark the correct answer with a cross.

12 cm^2^

7 cm

7 cm^2^

12 cm

14 cm

### Geometry task 4: “Puzzle pieces”

**Puzzle pieces**

**Which of these puzzle pieces has the largest surface area?**


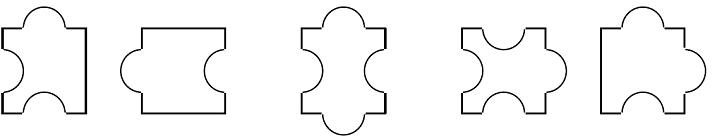
Mark the correct answer with a cross.

### Geometry task 5: “Neighboring sides in parallelogram”

**Neighboring sides in parallelogram**

In a parallelogram, one side is 40 cm long and an adjacent side is 90 cm long.

**What is the circumference of the parallelogram?**

Mark the correct answer with a cross.

130 cm

170 cm

260 cm

340 cm

360 cm

### Geometry task 6: “Thread feed”

**Thread feed**

A 34 cm long thread is laid to form a rectangle.
The width of the rectangle is 8 cm.

**How long is the rectangle?**

8 cm

9 cm

10 cm

13 cm

18 cm

### Geometry task 7: “Isosceles triangles” (4 items)

**Isosceles triangles**

**Are the following statements right or wrong?**

**Every isosceles triangle has …** right wrong

… three sides of equal length

… at least one axis of symmetry

… always a right angle

… at least two angles of equal size

### Geometry task 8: “Garden” (4 items)

**Garden**

A rectangular garden has a circumference of 100 m.

**How long and how wide can the garden be?**

right wrong

80 m long and 20 m wide

40 m long and 10 m wide

25 m long and 25 m wide

10 m long and 10 m wide

### Geometry task 9: “Cube volume”

**Cube volume**

**How does the volume of a cube change when the edge length is doubled?**

The volume …

... doubles

... quadruples

... increases sixfold

... increases eightfold

### Geometry task 10: “Cone” (4 items)

**Cone**

The sketched surfaces should be rotated in space around the dashed axes.

**At which surface is a cone created?**

Mark all possibilities.

surface A surface B surface C surface D


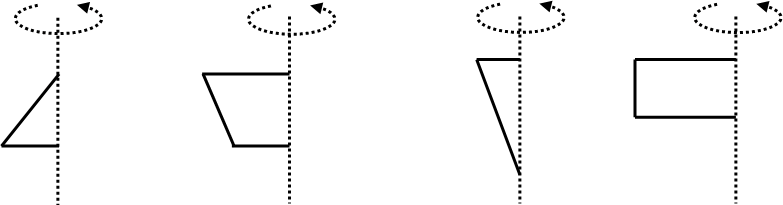


surface A surface B surface C surface D

yes yes yes yes

no no no no

## Stochastics (7 items)

### Stochastics task 1: “Tiles”

**Tiles**

One box contains 45 colored tiles: blue, green and yellow.

**If the probability of moving a yellow tile is** $\frac{\text{2}}{\text{5}}$**, how many yellow tiles are in the box?**

2

5

9

18

25

### Stochastics task 2: “Red-blue cube”

**Red-blue cube**

Each of the six surfaces of a cube is painted. Some surfaces are red, and some surfaces are blue. When throwing the dice, the probability that a red surface on top remains $\frac{\text{2}}{\text{3}}$.

**How many surfaces of the cube are painted red?**

1 surface

2 surfaces

3 surfaces

4 surfaces

5 surfaces

### Stochastics task 3: “Screws”

**Screws**

In a company where screws are manufactured, a final inspection is carried out at the end of the production process. An inspected box contains 10,000 screws. From this box 200 screws are randomly selected and checked. 10 of these screws were outside the standard.

**How many non-standard screws are there in the whole box?**

20 screws

50 screws

200 screws

500 screws

2,000 screws

### Stochastics task 4: “Grades” (4 items)

**Grades**

In Germany grades are given from 1 (= very good) to 6 (= insufficient). The pie chart shows the distribution of marks in an examination in English in a German school class.


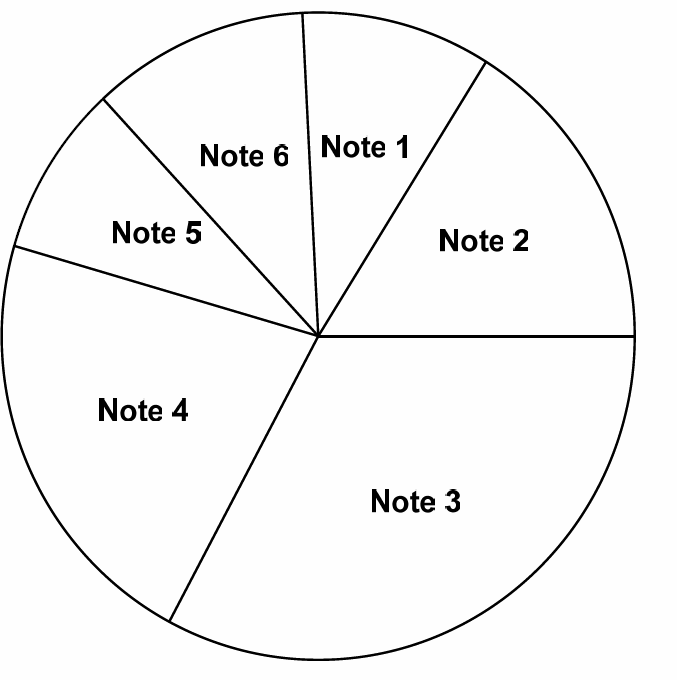


grade 1

grade 6

grade 5

grade 2

grade 4

grade 3

**Mark each statement with a cross, whether it is right or wrong**.

**Statement** right wrong

There is more often the grade 2 than the grade 4.

One third of the students have the grade 1 or the grade 2.

More than 50% of the students have a better grade than grade 4.

Less than a quarter of the students have the grade 3.

# Intelligence (*g*)

To cover general intelligence (g), we implemented 12 reasoning tasks of which four each referred to one of three statements. The students were asked to indicate which conclusions were mandatory. These statements with the corresponding conclusions are depicted in the following. Before that the general introductory instruction is shown.

**Conclusions**

What conclusions can be drawn with compelling necessity from a statement without any additional assumption?

Several conclusions can be correct in one finding.

**Decide for each conclusion whether it is right or wrong.**

## Intelligence task 1: “Vacations”

**Statement:***In Luxembourg travel agencies, more holiday trips are booked for the summer than for the winter.*

**Conclusion:**

correct      wrong

Luxembourg vacationers believe that they can relax better in summer than in winter.

The frequency with which vacations are booked in Luxembourg is not independent of the season.

More people in Luxembourg have vacations in summer than in winter.

Luxembourg vacationers tend to prefer to take their holidays in summer.

## Intelligence task 2: “Traffic”

**Statement:***The number of traffic accidents is increasing year by year.*

**Conclusion:**

correct      wrong

The road network is no longer adequate for today's traffic density.

Last year there were fewer road accidents than there will be next year.

The damage caused by traffic accidents is increasing year by year.

There are more cars today than there were ten years ago.

## Intelligence task 3: “Smoking”

**Statement:***Although the number of health problems acquired through smoking is increasing and health authorities repeatedly warn against smoking, cigarette consumption has continued to increase.*

**Conclusion:**

correct    wrong

Most smokers doubt that smoking leads to health problems.

The warnings issued by the health authorities have so far had no demonstrable effect on cigarette consumption.

Smoking is an addiction against which warnings are of no use.

Many people have so far preferred to accept damage to their health rather than giving up smoking.

# Reading literacy (*rl*)

In PROLOG-1, 4 tasks from the PISA 2006 reading test with several questions or subquestions each were implemented. These tasks with the corresponding questions are depicted in the following. For a full list of all published PISA 2006 items covering reading literacy (incl. the corresponding scoring), also see https://www.oecd.org/pisa/38709396.pdf.

## Reading task 1: “Lake Chad” (3 items)

**Lake Chad**

Figure 1 shows the fluctuations in the water level of Lake Chad in the Sahara in North Africa. During the last ice age, around 20,000 BC, Lake Chad disappeared completely. Around 11,000 BC, it was rebuilt. Today, it has the same water level as in 1,000 AD.


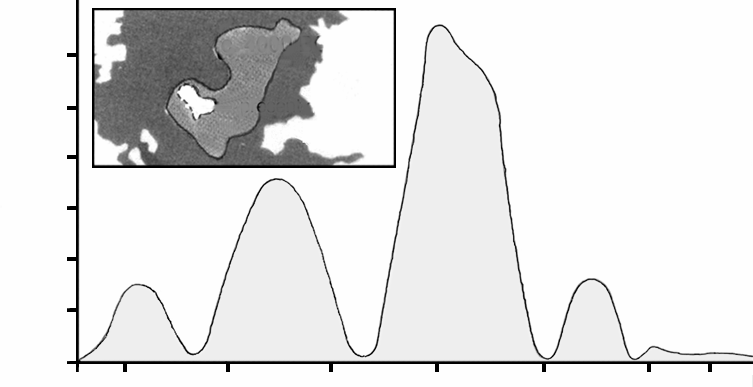


60

**c. 4000**

**BC**

50

**today**

Lake Chad:

Fluctuations of the water level

40

30

20

10

depth in meters

6,000 BC

10,000 BC

2,000 BC

8,000 BC

4,000 BC

Figure 1

0

Figure 2 shows rock paintings (old drawings or paintings found on the walls of caves) from the Sahara and changes in the structure of the animal world.


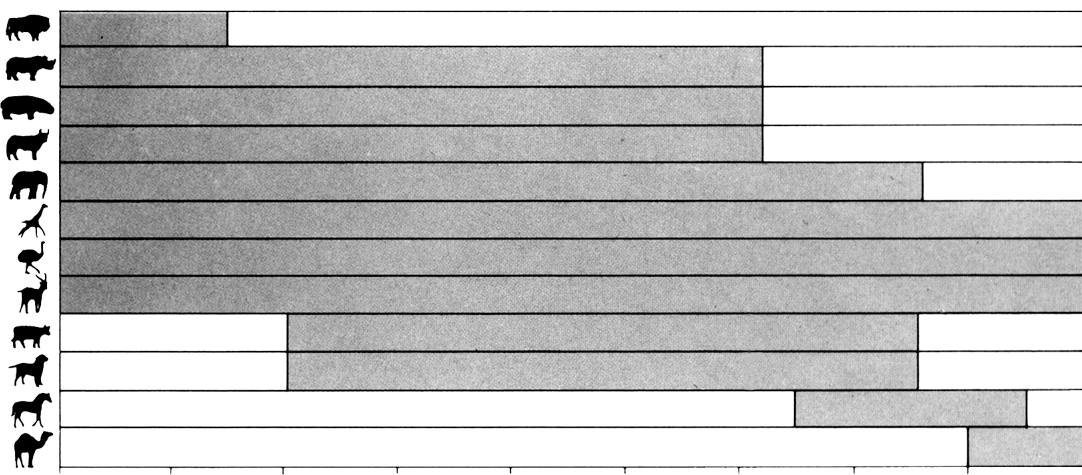

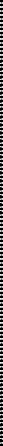


Rock paintings from the Sahara and changes in the structure of the animal world

Buffalo

Rhino

Hippo

Aurochs

Elephant

Giraffe

Ostrich

Gazelle

Cattle

Dog

Horse

Camel

8,000 7,000

BC BC

6,000 5,000

BC BC

4,000 3,000 2,000

BC BC BC

1,000

BC

0

1,000

AD

Figure 2

**Task 1:**

**How deep is Lake Chad today?**

About two metres.

About fifteen meters.

About 50 meters.

It has completely disappeared.

This information is not given.

**Task 2:**

**Figure 2 assumes that**

the animals in the rock paintings were present in the area at the time the paintings were created.

the artists who painted the animals were highly talented.

the artists who painted the animals could travel long distances.

there was no attempt to tame the animals depicted in the rock paintings.

**Task 3:**

For this question you have to combine information from figure 1 and figure 2.

**The disappearance of rhino, hippo and aurochs from the rock paintings in the Sahara happened**

at the beginning of the last ice age.

in the middle of the period when Lake Chad had its highest water level.

after Lake Chad's water level had been falling for tens of years.

at the beginning of an uninterrupted dry season.

## Reading task 2: “Flu” (3 items)

**A-COL VOLUNTARY INFLUENZA VACCINATION PROGRAM**

As you certainly know, the flu can spread quickly and widely in winter. Its victims then often lie in bed for weeks on end.


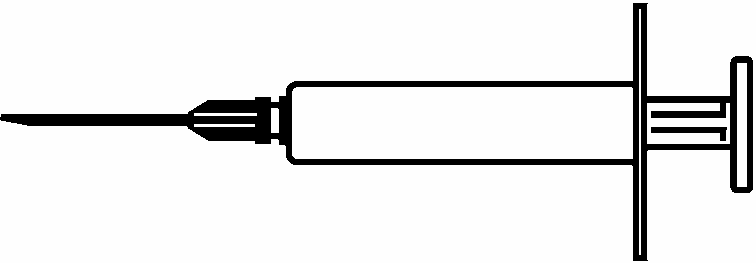
The best way to fight the virus is a fit and healthy body. Daily exercise and a diet with lots of fruit and vegetables are highly recommended to keep the immune system in its fight against this pathogen.

The ACOL company has decided to offer its employees the opportunity to offer flu vaccination as an additional way to prevent this insidious virus from spreading among us. ACOL has hired a nurse to administer the vaccinations at ACOL's premises for half a day during working hours during the week starting 17 November. This program is free of charge and is available to all employees.

Participation is voluntary. Employees who wish to take advantage of this opportunity are requested to sign a declaration of consent stating that they have no allergies and that they are aware that the vaccination may have minor side effects.

According to medical evidence, the vaccination does not cause flu infection. However, sometimes some side effects such as fatigue, mild fever and pain in the arm may occur.


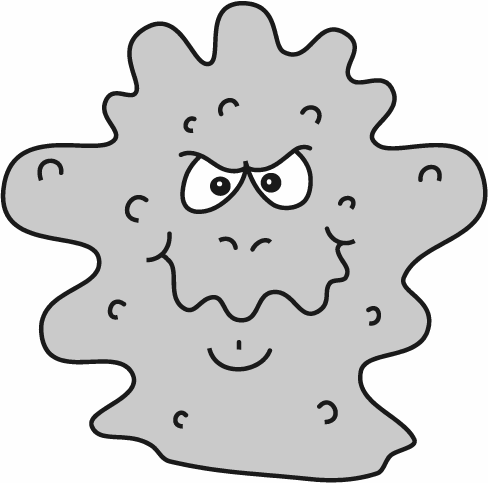


**WHO SHOULD GET VACCINATED?**

Anyone who wants to protect themselves from the virus.

This vaccination is especially recommended for people over 65. However, regardless of age, it is good for EVERYONE who suffers from a chronic debilitating disease, especially heart, lung, bronchial disease or diabetes.

In an office environment, ALL employees are at risk of catching the flu.

**WHO SHOULD NOT BE VACCINATED?**

People who are hypersensitive to eggs; people suffering from acute febrile illness and pregnant women.


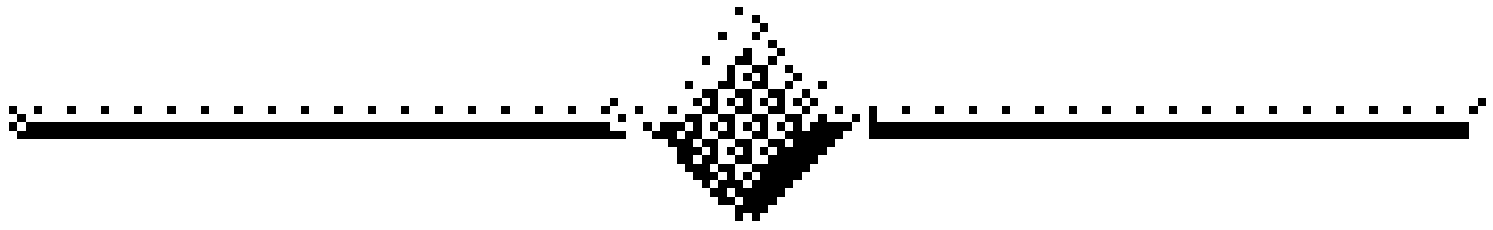
Ask your doctor if you are taking any medication regularly or if you have experienced side effects from a previous flu vaccination.

If you wish to be vaccinated in the week starting 17 November, please inform the personnel manager Renate Petersen by Friday, 7 November. The date and time will depend on the availability of the nurse, the number of participants and the most convenient time for most employees. If you would like to be vaccinated for this winter, but are unable to come at the appointed time, please inform Ms. Petersen. If there are enough participants, another appointment can be made.

For further information, please contact Ms. Petersen at extension 5577.

**Stay**

***healthy***

*Renate Petersen, Human Resources Manager at ACOL, has published the information sheet for ACOL employees printed on the previous two pages. Please refer to the information sheet for answers to the questions below.*

**Task 1:**

**Which of the following is part of ACOL's influenza vaccination program?**

During the winter, we offer daily sports activities.

Vaccinations are given during working hours.

Participants receive a small bonus.

The injections are given by a doctor.

**Task 2:**

**The information sheet states that if you want to protect yourself against the flu virus, a flu vaccination**

more effective than exercise and healthy food, but riskier.

a good thing, but no substitute for exercise and healthy eating.

just as effective as exercise and healthy food and less strenuous.

not necessary if you exercise a lot and eat a healthy diet.

**Task 3:**

**According to the information sheet, which of the following employees should contact Ms. Petersen?**

Mr. Siegert from the camp, who does not want to be vaccinated because he prefers to rely on his natural defences.

Mrs. Fischer from the sales department, who would like to know whether flu vaccination is compulsory.

Mrs. Albert from the dispatch department, who would like to be vaccinated this winter but will have a baby in two months.

Mr. Michaelis from the accounts department, who would like to be vaccinated but is on holiday in the week from 17 November.

## Reading task 3: “Labor” (8 items)

**Employed population**

The following tree diagram shows the structure of the working population or the “working age population” of a country. The total population of the country was about 3.4 million in 1995.

***The structure of the economically active population, as of 31 March 1995 (in thousands)^1^***

working age population^2^

**2,656.5**

Not employed

**128.1 7.5 %**

Employed

**1,578.4 92.5 %**

Not available to the labor market^3^

**949.9 35.8 %**

Available to the labor market

**1,706.5 64.2 %**

looking for full-time
employment

**318.1 93.2 %**

looking for full-time
employment

**23.2 6.8 %**

**341.3**

**78.4 %**

**1,237.1**

Full-time

looking for part-time
employment

**26.5 20.7 %**

looking for full-timeemployment

**101.6 79.3 %**

**21.6 %**

Part-time

**Notes**

1. number of persons in thousands (K).
2. the working-age population is defined as people between 15 and 65 years of age.
3. “Not available for the labor market” refers to persons who are not actively seeking work and/or are not available for work.

Use the information on the working population of a country on the opposite page to answer the questions below.

**Task 1:**

**Into which two main groups is the working age population divided?**

Employed and non-employed.

In working age and not in working age.

Full-time employees and part-time employees.

Available to the labor market and not available to the labor market.

**Task 2:**

**In which part of the tree diagram would the persons listed in the table below be included, if at all?**

Answer by checking the appropriate box in the table.

The first cross has already been made for you.

“Not available to the labor
market:”

“Available to the labor market: not employed”

“Available to the labor market:
employed”

Not
included in any category

A part-time waiter, 35 years

A businesswoman, 43 years old, with a 60-hour week

A full-time student, 21 years

A man, 28 years, who recently sold his property and is now looking for work

A woman, 55 years old, who never worked or wanted to work

A grandmother, 80 years old, who still works a few hours every day at her family's market stall

**Task 3:**

Imagine that this information about the structure of the working population would be published every year in a tree chart like this.

Below are four components of the tree chart.

**Indicate whether or not you would expect these components to change from year to year by placing a cross in the “Change” or “No change” column.**

The first cross has already been made for you.

**No**

Change

**Components of the tree diagram** Change

The inscription in each box (e.g. “Available to the labor market”)

The percentages (e.g. “64.2 %”)

The numbers (e.g. “2,656.5”)

The footnotes under the tree diagram

**Task 4:**

The information on the structure of the working population is presented in the form of a tree slide, but could have been presented in various other ways, such as a written description, a pie chart, a bar chart or a table.

**The tree diagram was probably chosen because it is particularly suitable for the representation of**

the changes over time.

in working age and not in working age.

the categories within each group.

the size of each group.

## Reading task 4: “Police” (4 items)


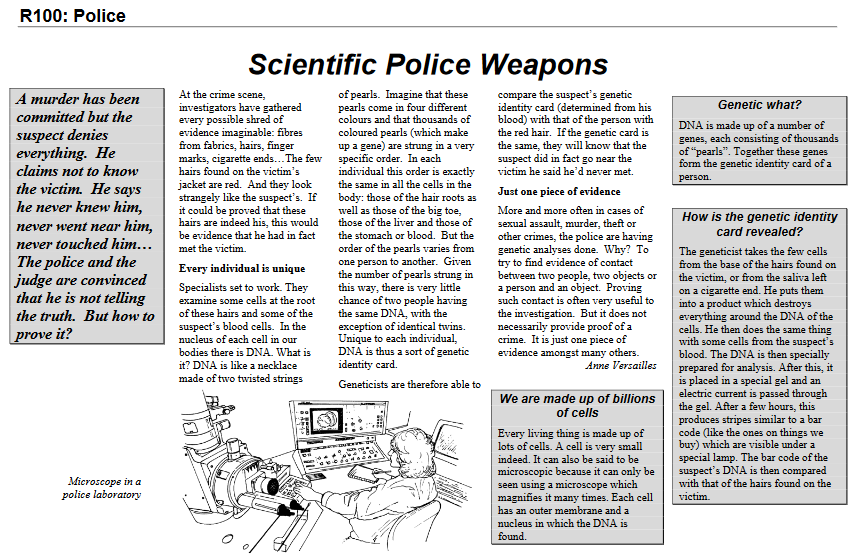


**Task 1:**

To explain the structure of DNA, the author speaks of a string of pearls.

**How do these pearl necklaces differ in different people?**

They are of different lengths.

The order of the pearls is different.

The number of chains is different.

The colour of the pearls is different.

**Task 2:**

**What is the purpose of the box with the title: “How to find the genetic Identity card?”**

It shall explain,

what the DNA is.

what a bar code is.

how cells are analyzed to find the pattern of DNA.

how to prove that a crime has been committed.

**Task 3:**

**What is the main objective of the author?**

To warn

To entertain

To inform

To convince

**Task 4:**

At the end of the introduction (first box with grey background) it says: “But how can it be proven?”

**The text says that investigators try to find an answer to this question by**

interviewing witnesses

performing genetic tests

thoroughly interrogate the suspect

go over all the results of the investigation again
